# Frequency and Associations of Adverse Reactions of COVID-19 Vaccines Reported to Pharmacovigilance Systems in the European Union and the United States

**DOI:** 10.3389/fpubh.2021.756633

**Published:** 2022-02-03

**Authors:** Diego Montano

**Affiliations:** Department of Population-Based Medicine, Institute of Health Sciences, University of Tübingen, Tübingen, Germany

**Keywords:** messenger RNA (mRNA), chimeric virus vaccines, SARS-CoV-2, pharmacovigilancce, mRNA vaccines

## Abstract

**Introduction:**

This study aims to provide a risk assessment of the adverse reactions related to the COVID-19 vaccines manufactured by AstraZeneca, Janssen, Moderna, and Pfizer-BioNTech which have been in use in the European Union and the United States between December 2020 and October 2021.

**Methods:**

Data from the European Database of Suspected Adverse Drug Reaction (EudraVigilance) and the Vaccine Adverse Events Reporting System (VAERS) from 2020 to October 2021 are analysed. More than 7.8 million adverse reactions of about 1.6 million persons are included. The adverse reactions are classified with the Common Toxicity Criteria (CTC) categories. COVID-19 vaccine exposures and adverse reactions reported between December 2020 and October 2021 are compared to influenza vaccine exposures and adverse reactions reported between 2020 and 2021. The population-level vaccine exposures to COVID-19 and influenza vaccines comprised about 451 million and 437 million exposures, respectively. Absolute and relative risk estimates are calculated by CTC categories and COVID-19 vaccines for the EU and US populations aged 18 years and older.

**Results:**

A higher risk of reporting serious adverse reactions was observed for the COVID-19 vaccines in comparison to the influenza vaccines. Individuals age 65 and older were associated with a higher frequency of death, hospitalisations, and life-threatening reactions than younger individuals (relative risk estimates between 1.49 99% CI [1.44–1.55] and 8.61 99% CI [8.02–9.23]). Outcome onset of serious adverse reactions occurred within the first 7 days after vaccination in about 77.6–89.1% of cases. The largest absolute risks were observed for allergic, constitutional reactions, dermatological, gastrointestinal, neurological reactions, and localised and non-localised pain. The largest relative risks between COVID-19 vs. influenza vaccines were observed for allergic reactions, arrhythmia, general cardiovascular events, coagulation, haemorrhages, gastrointestinal, ocular, sexual organs reactions, and thrombosis.

**Conclusion:**

The present study provides an overview of adverse reactions frequently reported to the pharmacovigilance systems following COVID-19 vaccination in the EU and US populations. Despite the limitations of passive reporting systems, these results may inform further clinical research investigating in more detail the pathophysiological mechanisms potentially associated with the COVID-19 vaccines.

## 1. Introduction

Between December 2020 and January 2021, the United States (US) Food and Drug Administration (FDA) and the European Medicines Agency (EMA) issued the so-called Emergency Use Authorizations and Conditional Marketing Authorisations, respectively, for the Pfizer-BioNTech and Moderna COVID-19 vaccines (1–4). The mRNA vaccines are products based on nucleic-acid pharmaceutical technology (5) and contain a nucleoside-modified messenger RNA (mRNA) encoding the viral spike S glycoprotein of SARS-CoV-2, lipid nanoparticles, and some salts, sugars, and buffers (6, 7). Besides the mRNA vaccines, two vectorised vaccines, Janssen COVID-19 and COVID-19 AstraZeneca vaccine (later re-branded as Vaxzevria) in the European Union (EU), have also received Emergency Use and Conditional Marketing Authorisations. The Janssen COVID-19 vaccine is a replication-defective human adenovirus type 26 (Ad26) vectored vaccine encoding the SARS-CoV-2 viral spike S glycoprotein (Ad26.COV2-S) (8). The COVID-19 AstraZeneca vaccine is a replication-defective chimpanzee adenovirus vectored vaccine encoding the SARS-CoV-2 viral spike S glycoprotein (ChAdOx1-S) (9). In general, the vector viruses of vectorised vaccines, also called “chimeric virus vaccines,” are genetically modified organisms obtained by standard recombinant DNA technology which genetically encode the target antigens (10, 11). The replication-defective adenovirus-vectored vaccines use the adenovirus backbone, a double-stranded DNA virus, to infect host cells which ultimately will express the SARS-CoV-2 viral spike S (12). In brief, the mode of action of the mRNA COVID-19 vaccines to induce an immune response against SARS-CoV-2 is based on the cellular internalisation of the lipid nanoparticles containing the mRNA encoding the spike S glycoprotein of SARS-CoV-2 which leads to the activation of antigen-presenting cells and ultimately to the production of immunoglobulin antibodies against the spike S protein (13). The mode of action of the chimeric virus vaccines is based on the ability of the chimeric adenovirus encoding the S glycoprotein of SARS-CoV-2 to infect human cells and induce the expression of the S spike protein resulting also in the development of antibodies against the S spike protein *via* antigen-presenting cells (12, 14).

Both the FDA and EMA require from vaccination providers or national health authorities to report adverse reactions such as vaccine administration errors or cases of hospitalisations and death to the Vaccine Adverse Event Reporting System (VAERS) and the European Database of Suspected Adverse Drug Reactions (EudraVigilance), respectively (6, 7, 15). In general, death, hospitalisation, life-threatening reactions, disabilities, and birth defects are defined as serious adverse outcomes. Several reasons make the ongoing mass vaccination programmes in the EU and US against SARS-CoV-2 unique: (i) Prior to 2021, there were no vaccines against coronaviruses approved for human use, (ii) most vectorised and mRNA-based vaccines were still in clinical research phases for the treatment of different cancer types, protein-replacement therapies, regenerative medicine, and vaccine development (16) and, (iii) similarly, there were few chimeric virus vaccines approved for human use, even though their application in oncology and veterinary practice was much more common (17, 18). In addition, both mRNA and vectorised COVID-19 vaccines have been authorised in a fast-track mechanism (FDA) or accelerated assessment procedure (EMA) (19, 20) and, therefore, as investigational new drugs, there are still uncertainties regarding the magnitude of their potential to elicit adverse reactions. Hence, the aim of this contribution is to identify potential safety issues of the new COVID-19 vaccines being currently deployed in the EU and US with data from the VAERS and EudraVigilance databases in the population age 18 years and older. In particular, this study aims to estimate the absolute and relative risks of reporting serious adverse reactions associated with the COVID-19 vaccines reports in comparison to influenza vaccines used during 2020 and 2021 in adult populations. In this manner, the present study contributes to pharmacovigilance research by providing a general overview of potentially causal relationships between vaccine exposure and reported adverse reactions which may be explored in future clinical studies assessing the extent to which some form of causal association can be inferred for particular adverse reactions. To the knowledge of the author, such an overview of adverse reactions with large pharmacovigilance datasets has not been published so far.

## 2. Data and Methods

### 2.1. Data

The EudraVigilance is a reporting system maintained by EMA which contains solicited and unsolicited suspected adverse reactions of pharmaceuticals for human use authorised in the EU. The adverse reaction reports in EudraVigilance come from cases within the EU and the European Economic Area (EEA) submitted by national health authorities and the marketing authorisation holders (21). The medical conditions of cases reported to Eudravigilance are coded by using the Medical Dictionary for Regulatory Activities (MedDRA) terms at their lowest coding level (22). The MedDRA terms do not represent medical diagnoses, but simply the encoding of reported adverse events in a structured classification system of medical conditions. The individual case safety reports in the EudraVigilance database are subjected to a validation process involving the competent authorities in the EU Member States and the marketing authorisation holders (22, p. 82f.). In the present investigation, data from EudraVigilance from 2020 to 18 October 2021 are included in the analyses. A total of 4,173,937 reactions of 1,096,569 persons age 18 and older are included. For each reported case there can be an unlimited amount of MedDRA-coded terms and, thus, multiple rows per reported case. The datasets from the EudraVigilance system are publicly available and can be obtained by querying the line listings view of adverse reactions for each vaccine type and reporting year.

The Vaccine Adverse Event Reporting System (VAERS) is a passive reporting system on post-licence safety monitoring of US licensed vaccines (23). Information collected in VAERS is passively received from those who choose to voluntarily report an adverse event following immunisation. The reported cases may include not only reactions directly associated with the vaccine (e.g., pain at the injection site), but also quality defects or vaccination administration errors (e.g., storage issues) (23). Healthcare professionals, vaccine providers and manufacturers, patients, parents, caregivers, or others can report an adverse event to VAERS. The adverse reactions in VAERS are also coded by using the MedDRA terms and, thus, they allow a direct comparison of reactions between different surveillance systems (24). In the present investigation, reports from 2020 to 10 October 2021 are included. A total of 3,651,010 reactions of 534,332 persons age 18 years and older are included. Each reported case in VAERS may include more than one adverse reaction, just as in the EudraVigilance database. The raw data files from VAERS can be obtained by downloading the ZIP files made available on the VAERS website for each reporting year.

Data on weekly COVID-19 vaccine coverage in the EU and the European Economic Area (EEA) (week 51/2020 to week 42/2021) and the US (week 51/2020 to week 43/2021) are publicly available from the European Centre for Disease and Prevention (ECDC) (25) and the Centers for Disease and Prevention (CDC) (26), respectively. In the present study, the number of individuals age 18 and older having received the first dose of either AstraZeneca, Janssen, Moderna, or Pfizer-BioNTech in one of the EU and EEA countries and the US were considered. The following influenza vaccine types were included: monovalent, trivalent, or quadrivalent split virion and surface antigen influenza vaccines produced mainly by GlaxoSmithKline, Pfizer/Seqirus, AstraZeneca, Abbot Biologicals, Sanofi Pasteur, and Mylan Products (27). The sources of all the datasets used in the present analyses are provided in the Data Availability section.

### 2.2. Classification of Adverse Reactions

Although the different MedDRA coding levels used in VAERS and EudraVigilance allow a relatively detailed description of the particular medical conditions mentioned in the reports, it is necessary to take into account the different biological pathways linking vaccine exposure and adverse reaction. To this end, the medical conditions coded in VAERS and EudraVigilance are classified in 17 event categories following the Common Toxicity Criteria (CTC) developed by the National Cancer Institute in the US, which is one of the oldest and most commonly used classification systems of adverse reactions in clinical trials (28). The CTC classification groups adverse reactions according to pathophysiological and anatomical categories and provide a more adequate identification of the potential biological mechanisms responsible for the reported adverse reactions. The categories are defined very broadly and include any unfavourable symptom, sign, disease, or abnormal laboratory finding temporally associated with the use of a medical treatment (28). Notwithstanding the generic character of the definition of the single CTC categories, they allow a more clinically meaningful interpretation of results. The event categories considered in the present investigation correspond to the following major categories: allergic/immunologic reactions (e.g., drug pyrexia, pruritus, urticaria), cardiovascular events related to arrhythmia, haematological reactions (e.g., lymphopenia, abnormal neutrophil count), general cardiovascular events (e.g., myocardial infarction, hypertension, myocarditis, pericarditis), coagulation (e.g., disseminated intravascular coagulation, abnormal platelet count), thrombotic reactions, constitutional symptoms (e.g., fatigue, lethargy, malaise), dermatological (e.g., erythema), gastrointestinal (e.g., diarrhoea), haemorrhage (excluding sexual organs, e.g., cerebral haemorrhage, adrenal haemorrhage, petechiae), neurological reactions (e.g., aphasia, dizziness, ataxia, seizures, tremor), ocular, localised pain (e.g., injection site pain), non-localised pain (e.g., abdominal pain, arthralgia, axillary pain, myalgia), pulmonary (e.g., apnoea, dyspnoea), renal/genitourinary and sexual organs (including haemorrhages, e.g., ovarian and penile haemorrhage). The complete list of medical conditions in each CTC event category is provided in the Supplementary Material.

### 2.3. Statistical Analysis

One of the major drawbacks of spontaneous reports of adverse reactions is the fact that the calculation of risk differences needed in causal inference is not straightforward due to under- or over-reporting of adverse reactions, non-ignorable treatment assignment processes and uncertainties regarding the number of individuals exposed to the vaccines (29). In the case of the ongoing COVID-19 vaccination programmes in the EU and US, however, two circumstances allow the calculation of unbiased risk estimators of adverse reactions for the COVID-19 vs. influenza vaccines, namely: (1) the number of individuals exposed to the COVID-19 vaccines and the age distribution are known and can be used as a denominator to calculate unbiased risk estimates for COVID-19 vaccines (29) and (2) data on adverse reactions related to the influenza vaccines provide an ideal control group for COVID-19 vaccination, since vaccine platforms based on nucleic acid technology had never been deployed for prophylactic vaccination of the general population prior to the emergence of the novel SARS-CoV-2 in December 2019 in China. In addition, the number of individuals vaccinated against influenza has been well documented, especially in the US where the CDC provides for each season weekly estimates of influenza vaccination. Moreover, influenza vaccination represents an ideal control for the ongoing COVID-19 vaccination due to the fact that, on the one hand, seasonal influenza-viruses share with coronaviruses substantial similarities regarding symptomatology, infectivity, pathogenecity, letality, and transmission and, on the other hand, a large proportion of the adult populations in the EU and US is vaccinated against influenza every season. The rationale behind the comparison of adverse reaction risks between COVID-19 and influenza vaccines is to assess the potential risk profile of the new nucleic-acid-based pharmaceutical platforms in comparison to the traditional vaccination platforms based on live, inactivated or attenuated pathogens or immunoglobulins. Thus, COVID-19 vaccines and influenza vaccines are not compared by their mode of action, but on the common metric of the probability of observing serious adverse reactions following vaccination.

In the present study the risk of adverse reactions for COVID-19 vaccines (*R*_*c*_), influenza vaccines (*R*_*n*_), the corresponding relative risks (*RR*), and their variance (Var) are calculated as follows (30):


(1) $$\begin{array}{l} R_{c}=\frac{K_{c}}{c}\;\text{and}\;R_{n}=\frac{K_{n}}{n} \end{array}$$

(2) $$\begin{array}{l} RR=\frac{R_{c}}{R_{n}}\;\text{with}\;\text{Var}\left(\log RR\right)=\frac{1-K_{c}/c}{K_{c}}+\frac{1-K_{n}/n}{K_{n}} \end{array}$$

where the numerators *K*_*c*_ and *K*_*n*_ represent the number of adverse reaction in each CTC event category for COVID-19 and influenza vaccines, and the denominators *c* and *n* correspond to the estimated number of individuals 18 years and older vaccinated with COVID-19 and influenza vaccines, respectively. Please note that the numerator *K*_*n*_ does not contain reports of individuals 17 and younger, but only adults aged 18 years and older. The denominator of the COVID-19 vaccine risks *R*_*c*_ corresponds to the total number of individuals older than 18 years having received at least one dose of one of the COVID-19 vaccines in the EU and US. By end October 2021, approximately 246,534,547 and 205,482,061 persons age 18 and older have received at least one COVID-10 vaccine dose, according to the official data from the ECDC and CDC in both the EU and US (ECDC and CDC) (25, 26).

The denominators of influenza vaccines in the EU and the US correspond to about 77.1 and 361 million influenza doses for the last two influenza seasons between 2020 and 2021, respectively. The US estimates were obtained from the official statistics provided by the CDC on a weekly and seasonal basis (31). For the EU, unfortunately, there are no weekly and seasonal statistics for the Member States. Nonetheless, the European Statistical Office Eurostat provides in the variable *hlth_ps_immu_esms_an1* for the influenza season 2018–2019 estimates of influenza vaccination coverage for the population 65 years and older which can be used as a lower bound estimate of the number of influenza exposures expected for the last two influenza seasons 2020–2021 (32). Thus, given an influenza vaccination coverage of 42% for the 2018–2019 season and a total population 65 and older in the EU27 of approximately 91.85 million in the year 2020 (32), for the last two influenza seasons 2020-2021 at least 77.1 million influenza vaccine exposures can be expected.

The estimation of the adverse reaction risks for each COVID-19 vaccine manufacturer, namely, AstraZeneca, Janssen, Moderna, and Pfizer-BioNTech, follows Equation (1). The numerator is the total number of adverse reactions for each COVID-19 vaccine product, whereas the denominator corresponds to the estimated number of individuals who have received at least one dose of the corresponding product, as expected on the basis of the proportion of administered doses by vaccine product (25, 26). Similarly, the calculation of adverse reaction risks for sex (males and females) and two age groups (18-64 and older than 65 years) is based on Equation (1), where the denominators represent the total number of individuals who have received at least one COVID-19 vaccine doses in each sex and age category. By taking the actual number of exposed individuals in each age group, it is possible to adjust the risk estimates for the fact that the vaccination programmes in the US and EU started with the vaccination of older individuals and, thus, the vaccination coverage in the elderly is greater than for individuals younger than 65. The proportion of male and female individuals vaccinated against COVID-19 is very similar in the US and the EU and, for the sake of comparability, is set at 50% for both the US and the EU. The reported confidence intervals were estimated at the 99% level to reduce the probability of false positives for small effects in large samples. Data preparation and statistical analyses were performed with the statistical environment R v.3.6.

## 3. Results

**Figure 1:**
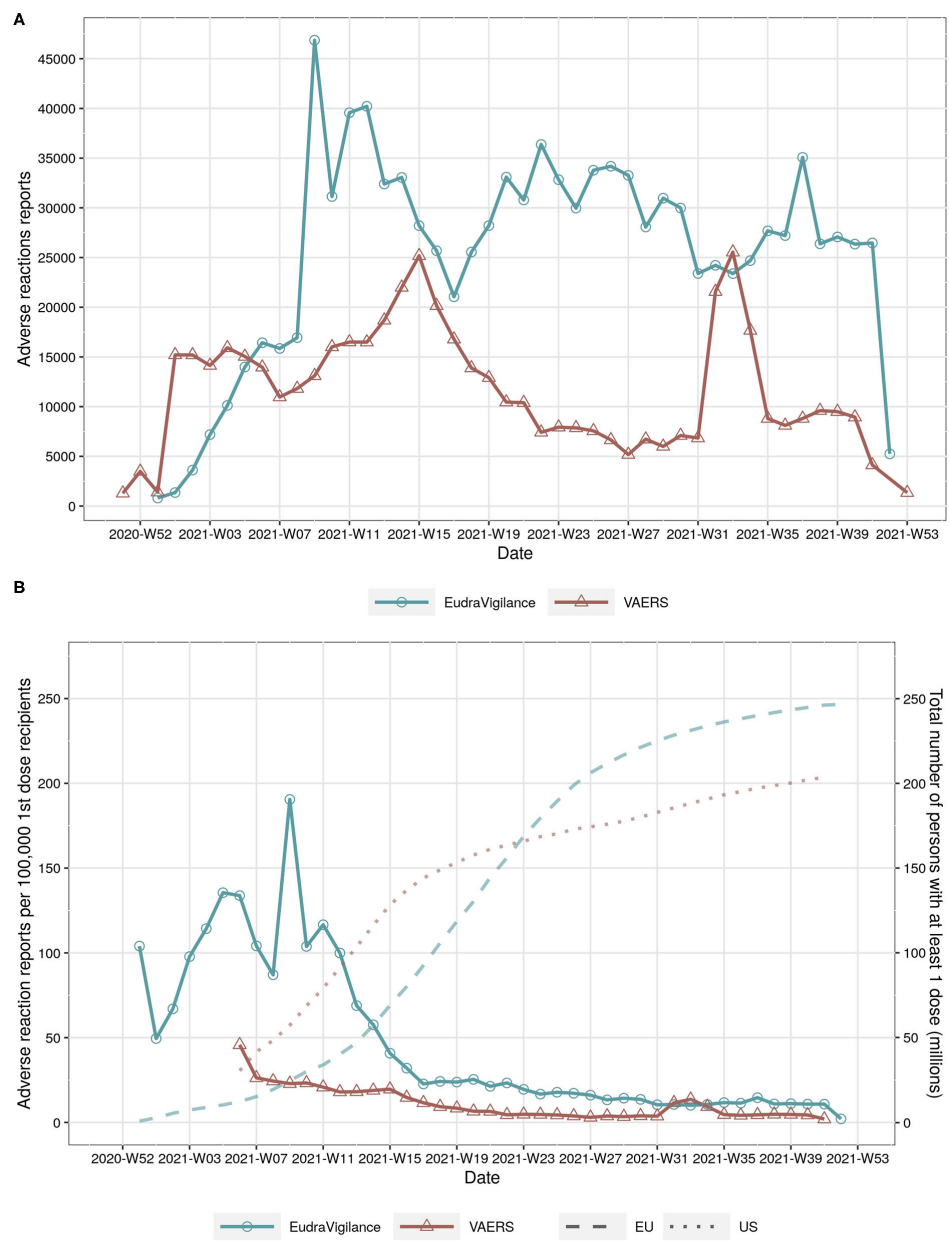
**(A)** total number of adverse reactions reports in EudraVigilance and VAERS by year and week. **(B)** number of adverse reactions reports per 100,000 persons with at least 1 COVID-19 vaccine dose (continuous line, left y-axis) and total number of persons with at least 1 dose in the EU and US by year and week (dotted lines on background, right y-axis).

The time series of the absolute number of cases with adverse reactions reported to EudraVigilance and VAERS show large variations over time, with the number of reports peaking at about weeks 8, 15, 33, and 37 in 2021 (Figure 1A). However, the total number of reports per week in EudraVigilance is substantially larger in comparison to VAERS, in particular since mid April 2021. The differences between both reporting systems are even more pronounced if the time series of the total number of persons aged 18 and older with at least 1 dose in the EU and US are compared (Figure 1B). Even though the vaccination coverages in the US and EU are comparable during the observation period (dotted lines and right y-axis in Figure 1B), the number of reports to VAERS per 100,000 first-dose recipients has been lower than in EudraVigilance, in particular until August 2021 (continuous lines and left y-axis in Figure 1B).

The absolute and relative risk estimates of reporting COVID-19 vaccine deaths, hospitalisations, and life-threatening outcomes are reported in Table 1. Outcome onset occurred within the first 7 days after vaccination in about 77.6–89.1% of cases related to deaths, hospitalisations, and life-threatening outcomes in the EudraVigilance reports. Similar estimates were obtained for the VAERS reports in which about 80.5% to 82.7% of cases with serious adverse reactions fall within 7 days after COVID-19 vaccine exposure. In both EudraVigilance and VAERS, higher absolute risks of reporting serious outcomes were observed among individuals age 65 and older (Table 1). The estimated relative risks (*RR*) for death reports between younger and older individuals indicate a higher probability of reported deaths among the elderly, especially for males. The absolute risks of deaths, hospitalisations, and life-threatening reactions for each COVID-19 vaccine product in EudraVigilance suggest that the Moderna COVID-19 vaccine is more frequently related to those serious outcomes in comparison to Janssen's vaccine. In VAERS, however, there were no large differences between the different manufacturers in comparison to Janssen (Table 1). Taken together, the COVID-19 vaccines are associated with higher absolute risks of serious adverse outcomes in comparison to influenza vaccines used in 2020 and 2021. The same association pattern is observed for the overall relative risks (*RR*), even though the corresponding estimates are usually larger in the EudraVigilance report system than in VAERS.

**Table 1:** Vaccine-related risk estimates (*R*) of serious outcomes per 100,000 exposed individuals, relative risk estimates (*RR*) and 99% confidence intervals (CI) in EudraVigilance and VAERS databases.

		**EudraVigilance**				**VAERS**			
		**Exposed**	**Cases**	**R**	**RR 99% CI**	**Exposed**	**Cases**	**R**	**RR 99% CI**
**Death cases**									
Age^a^, ^b^	18–64 Years	183,061,142	3,759	2.053	Ref.	15,2704,862	1,781	1.166	Ref.
	More than 65 Years	63,473,405	11,209	17.659	8.60 [8.19–9.03]	52,777,199	5,297	10.037	8.61 [8.02–9.23]
Sex	Female	123267273.5	7,202	5.843	Ref.	102,741,030	3,029	2.948	Ref.
	Male	123267273.5	8,330	6.758	1.16 [1.11–1.21]	10,2741,030	3,970	3.864	1.31 [1.23–1.39]
COVID vaccine	Astra	34,643,783	3,574	10.316	2.09 [1.88–2.32]				
	Janssen	14,723,578	727	4.938	Ref.	17,509,539	636	3.632	Ref.
	Moderna	24957523	3680	14.745	2.99 [2.69–3.32]	80561024	3238	4.019	1.11 [0.99–1.24]
	Pfizer	172,209,328	7,929	4.604	0.93 [0.84–1.03]	107,411,499	3204	2.983	0.82 [0.73–0.92]
All COVID vaccines		246,534,547	15,910	6.453	42.53 [33.49–54.01]	205,482,061	7,078	3.445	345.42 [224.61–531.20]
Influenza vaccines		7.71e+07	117	0.152	Ref.	3.61e+08	36	0.01	Ref.
**Hospitalisations**									
Age	18–64 Years	183,061,142	54,786	29.928	Ref.	15,2704,862	16,990	11.126	Ref.
	More than 65 Years	63,473,405	33,872	53.364	1.78 [1.75–1.82]	52,777,199	16,799	31.83	2.86 [2.78–2.94]
Sex	Female	123267273.5	51,885	42.091	Ref.	102,741,030	17,862	17.385	Ref.
	Male	123267273.5	39,879	32.352	0.77 [0.76–0.78]	10,2741,030	15,812	15.39	0.89 [0.86–0.91]
COVID vaccine	Astra	34,643,783	25,453	73.471	2.56 [2.45–2.67]				
	Janssen	14,723,578	4,231	28.736	Ref.	17,509,539	3,670	20.96	Ref.
	Moderna	24,957,523	19,864	79.591	2.77 [2.65–2.89]	80561024	13628	16.916	0.81 [0.77–0.85]
	Pfizer	172209328	43420	25.214	0.88 [0.84–0.91]	107,411,499	16,491	15.353	0.73 [0.70–0.77]
All COVID vaccines		246534547	92968	37.71	45.71 [41.26–50.65]	205,482,061	33,789	16.444	189.65 [163.85–219.53]
Influenza vaccines		7.71e+07	636	0.825	Ref.	3.61e+08	313	0.087	Ref.
**Life-threatening reactions**									
Age	18–64 Years	18,3061,142	13,997	7.646	Ref.	152,704,862	57,07	3.737	Ref.
	More than 65 Years	63,473,405	7,248	11.419	1.49 [1.44–1.55]	52,777,199	3,139	5.948	1.59 [1.50–1.69]
Sex	Female	123267273.5	12,122	9.834	Ref.	102741030	4858	4.728	Ref.
	Male	123267273.5	9785	7.938	0.81 [0.78–0.84]	102,741,030	3,958	3.852	0.81 [0.77–0.86]
COVID vaccine	Astra	34,643,783	7,534	21.747	2.75 [2.54–2.99]				
	Janssen	14723578	1163	7.899	Ref.	17509539	1108	6.328	Ref.
	Moderna	249,57,523	4,336	17.374	2.20 [2.02–2.39]	80,561,024	3535	4.388	0.69 [0.63–0.76]
	Pfizer	172,209,328	9,221	5.355	0.68 [0.63–0.73]	107,411,499	4,203	3.913	0.62 [0.57–0.67]
All COVID vaccines		246,534,547	22,254	9.027	56.13 [44.51–70.78]	205,482,061	8,846	4.305	196.72 [147.04–263.19]
Influenza vaccines		7.71e+07	124	0.161	Ref.	3.61e+08	79	0.022	Ref.

*Ref: Reference category for the estimation of the relative risks*.

a *The number of exposed individuals aged 18 to 64 years in the European Union corresponds to the interval 18–69 years since the official EU statistics on vaccination coverage contains only 10-year age intervals*.

b *Germany, Liechtenstein and Netherlands supply total numbers only. Based on national health authorities estimates, the number of exposed individuals in the 18–69 age interval is about 60%*.

The absolute and relative risk estimates by CTC categories and COVID-19 vaccine manufacturer in the EudraVigilance reports are provided in Tables 2a,b. The largest absolute risks in the EU were observed for allergic, constitutional reactions, dermatological, gastrointestinal, neurological, and localised and non-localised pain, especially concerning the AstraZeneca and Pfizer-BioNTech vaccines (Table 2a). However, the relative risks of the COVID-19 vaccines in comparison to influenza vaccines yielded large relative risks of allergic reactions, arrhythmia, general cardiovascular events, coagulation, haemorrhages, constitutional, gastrointestinal, ocular, sexual organs reactions, and, in particular, thrombosis (Table 2b). The largest relative risks in EudraVigilance were observed for AstraZeneca, Moderna, and Pfizer-BioNTech. Concerning the VAERS reports, a similar pattern of absolute risks across CTC categories can be observed, with allergic, constitutional reactions, dermatological, gastrointestinal, neurological, and non-localised pain accounting for the most frequently reported reactions, especially for Moderna and Pfizer-BioNTech (Table 3a). In agreement with the results obtained with EudraVigilance data, the relative risks calculated with VAERS data were also larger for allergic reactions, arrhythmia, general cardiovascular events, coagulation, haemorrhage, ocular, sexual organs reactions, and especially thrombosis for all COVID-19 vaccines (Table 3b).

**TABLE 2a:** Vaccine-related risk estimates of influenza (*R*_*n*_) and COVID-19 vaccines (*R*_*c*_) per 100,000 exposed individuals by Common Toxicity Criteria (CTC) in the EudraVigilance database.

			**AstraZeneca**		**Janssen**		**Moderna**		**Pfizer-BioNTech**	
**CTC**	**Influenza cases**	***R*_*n*_**	**Cases**	***R*_*c*_**	**Cases**	***R*_*c*_**	**Cases**	***R*_*c*_**	**Cases**	***R*_*c*_**
Allergic	1941	2.52	172092	69.80	12216	4.96	51360	20.83	134015	54.36
Arrythmia	235	0.30	20614	8.36	1690	0.69	11438	4.64	32245	13.08
Haematological	8	0.01	179	0.07	88	0.04	52	0.02	414	0.17
Cardiovascular	159	0.21	14328	5.81	2102	0.85	8959	3.63	27679	11.23
Coagulation	13	0.02	3392	1.38	673	0.27	532	0.22	2191	0.89
Constitutional	1392	1.81	260666	105.73	30361	12.32	73477	29.80	223516	90.66
Dermatological	1966	2.55	31509	12.78	4534	1.84	39148	15.88	47936	19.44
Gastrointestinal	848	1.10	103489	41.98	7684	3.12	29897	12.13	99231	40.25
Haemorraghe	50	0.06	4942	2.00	407	0.17	1504	0.61	4850	1.97
Neurological	1144	1.48	93643	37.98	6680	2.71	29240	11.86	103704	42.06
Ocular	122	0.16	15325	6.22	951	0.39	3976	1.61	13463	5.46
Localised pain	990	1.28	47475	19.26	4936	2.00	24161	9.80	79290	32.16
Non-localised pain	2225	2.89	251668	102.08	21036	8.53	69183	28.06	246484	99.98
Pulmonary	455	0.59	24134	9.79	2550	1.03	14101	5.72	40103	16.27
Renal/Genitourinary	26	0.03	816	0.33	142	0.06	877	0.36	1497	0.61
Sexual organs	9	0.01	9926	4.03	1277	0.52	5013	2.03	26667	10.82
Thrombosis	17	0.02	11254	4.56	2239	0.91	3257	1.32	8150	3.31
Other reactions	8450	10.96	577904	234.41	53467	21.69	216670	87.89	683218	277.13

*Denominators of R_n_ and R_c_: 7.71 × 10^7^ and 246,534,547 exposed individuals age 18 and older, respectively. Only the last two decimals of the risk estimates are reported*.

**TABLE 2b:** Vaccine-related relative risk estimates (RR) and 99% confidence intervals (CI) by Common Toxicity Criteria (CTC) in the EudraVigilance database.

	**AstraZeneca**	**Janssen**	**Moderna**	**Pfizer-BioNTech**
**CTC**	**RR 95% CI**	**RR 95% CI**	**RR 95% CI**	**RR 95% CI**
Allergic	27.73 [26.14–29.41]	1.97 [1.85–2.10]	8.28 [7.80–8.78]	21.59 [20.36–22.90]
Arrythmia	27.43 [23.17–32.48]	2.25 [1.88–2.69]	15.22 [12.85–18.04]	42.91 [36.25–50.79]
Haematological	7.00 [2.76–17.75]	3.44 [1.33–8.91]	2.03 [0.76–5.41]	16.18 [6.45–40.59]
Cardiovascular	28.18 [22.95–34.61]	4.13 [3.35–5.11]	17.62 [14.34–21.65]	54.44 [44.36–66.82]
Coagulation	81.60 [39.89–166.93]	16.19 [7.87–33.30]	12.80 [6.21–26.37]	52.71 [25.75–107.91]
Constitutional	58.56 [54.65–62.76]	6.82 [6.36–7.32]	16.51 [15.40–17.70]	50.22 [46.86–53.82]
Dermatological	5.01 [4.72–5.32]	0.72 [0.67–0.77]	6.23 [5.87–6.61]	7.63 [7.19–8.09]
Gastrointestinal	38.17 [34.92–41.71]	2.83 [2.58–3.11]	11.03 [10.08–12.06]	36.60 [33.48–40.00]
Haemorraghe	30.91 [21.43–44.58]	2.55 [1.73–3.74]	9.41 [6.50–13.62]	30.34 [21.03–43.75]
Neurological	25.60 [23.71–27.64]	1.83 [1.68–1.98]	7.99 [7.40–8.64]	28.35 [26.26–30.61]
Ocular	39.28 [31.08–49.65]	2.44 [1.90–3.12]	10.19 [8.04–12.91]	34.51 [27.30–43.62]
Localised pain	15.00 [13.81–16.29]	1.56 [1.43–1.71]	7.63 [7.02–8.30]	25.05 [23.07–27.20]
Non-localised pain	35.37 [33.49–37.37]	2.96 [2.79–3.13]	9.72 [9.20–10.28]	34.64 [32.80–36.60]
Pulmonary	16.59 [14.68–18.74]	1.75 [1.54–2.00]	9.69 [8.57–10.96]	27.56 [24.41–31.12]
Renal/Genitourinary	9.82 [5.88–16.40]	1.71 [0.99–2.96]	10.55 [6.32–17.61]	18.01 [10.82–29.97]
Sexual organs	344.91 [146.10–814.27]	44.37 [18.75–105.03]	174.19 [73.76–411.39]	926.63 [392.61–2187.06]
Thrombosis	207.03 [110.79–386.86]	41.19 [22.00–77.11]	59.92 [32.03–112.09]	149.93 [80.22–280.21]
Other reactions	21.39 [20.79–22.00]	1.98 [1.92–2.04]	8.02 [7.79–8.25]	25.29 [24.58–26.01]

**TABLE 3a:** Vaccine-related risk estimates of influenza (*R*_*n*_) and COVID-19 vaccines (*R*_*c*_) per 100,000 exposed individuals by Common Toxicity Criteria (CTC) in the VAERS database.

			**Janssen**		**Moderna**		**Pfizer-BioNTech**	
**CTC**	**Influenza cases**	***R*_*n*_**	**Cases**	***R*_*c*_**	**Cases**	***R*_*c*_**	**Cases**	***R*_*c*_**
Allergic	3197	0.89	15597	7.59	119339	58.08	71438	34.77
Arrythmia	384	0.11	3884	1.89	12431	6.05	15476	7.53
Haematological	16	0.00	251	0.12	929	0.45	1012	0.49
Cardiovascular	116	0.03	2291	1.11	8747	4.26	10193	4.96
Coagulation	32	0.01	1071	0.52	2102	1.02	2422	1.18
Constitutional	1925	0.53	20242	9.85	91552	44.55	74901	36.45
Dermatological	3120	0.86	2844	1.38	70249	34.19	18060	8.79
Gastrointestinal	1447	0.40	11916	5.80	51404	25.02	47570	23.15
Haemorraghe	42	0.01	393	0.19	1258	0.61	1476	0.72
Neurological	2249	0.62	16985	8.27	60221	29.31	69858	34.00
Ocular	329	0.09	2440	1.19	7633	3.71	8710	4.24
Localised pain	1298	0.36	2922	1.42	27747	13.50	14459	7.04
Non-localised pain	5281	1.46	27614	13.44	131636	64.06	114852	55.89
Pulmonary	611	0.17	6282	3.06	24586	11.97	29674	14.44
Renal/Genitourinary	23	0.01	277	0.13	1297	0.63	1481	0.72
Sexual organs	17	0.00	939	0.46	3347	1.63	5540	2.70
Thrombosis	6	0.00	1426	0.69	2252	1.10	2656	1.29
Other reactions	41992	11.63	217461	105.83	1092460	531.66	1055122	513.49

*Denominators of R_n_ and R_c_: 3.61 × 10^8^ and 205, 482, 061 exposed individuals, respectively. Only the last two decimals of the risk estimates are reported*.

**TABLE 3b:** Vaccine-related relative risk estimates (RR) and 99% confidence intervals (CI) by Common Toxicity Criteria (CTC) in the VAERS database.

	**Janssen**	**Moderna**	**Pfizer-BioNTech**
**CTC**	**RR 95% CI**	**RR 95% CI**	**RR 95% CI**
Allergic	8.57 [8.15–9.01]	65.58 [62.62–68.68]	39.26 [37.47–41.13]
Arrythmia	17.77 [15.48–20.39]	56.87 [49.77–64.99]	70.80 [61.98–80.88]
Haematological	27.56 [14.19–53.55]	102.01 [53.28–195.30]	111.12 [58.07–212.65]
Cardiovascular	34.70 [27.15–44.34]	132.48 [104.13–168.53]	154.38 [121.37–196.35]
Coagulation	58.80 [37.04–93.34]	115.40 [72.94–182.59]	132.97 [84.08–210.29]
Constitutional	18.47 [17.37–19.64]	83.55 [78.74–88.66]	68.36 [64.41–72.55]
Dermatological	1.60 [1.50–1.71]	39.56 [37.74–41.47]	10.17 [9.67–10.69]
Gastrointestinal	14.47 [13.47–15.54]	62.41 [58.27–66.85]	57.76 [53.92–61.87]
Haemorraghe	16.44 [10.82–24.97]	52.62 [35.13–78.82]	61.74 [41.26–92.39]
Neurological	13.27 [12.52–14.06]	47.04 [44.51–49.72]	54.57 [51.64–57.67]
Ocular	13.03 [11.20–15.16]	40.76 [35.26–47.12]	46.51 [40.25–53.75]
Localised pain	3.95 [3.63–4.31]	37.56 [34.91–40.41]	19.57 [18.16–21.09]
Non-localised pain	9.19 [8.84–9.55]	43.79 [42.24–45.40]	38.21 [36.85–39.62]
Pulmonary	18.06 [16.20–20.15]	70.69 [63.62–78.56]	85.32 [76.80–94.80]
Renal/Genitourinary	21.16 [12.10–37.00]	99.07 [57.63–170.32]	113.13 [65.84–194.37]
Sexual organs	97.04 [51.66–182.27]	345.89 [184.90–647.06]	572.52 [306.24–1070.35]
Thrombosis	417.54 [145.56–1197.72]	659.40 [230.06–1889.96]	777.70 [271.39–2228.54]
Other reactions	9.10 [8.97–9.22]	45.71 [45.12–46.30]	44.14 [43.58–44.71]

Even though a detailed presentation of the risk estimates for specific reactions is not feasible in the main manuscript due to the large number of specific reactions within each CTC category, interested readers are referred to the tables included in the Supplementary Material which provide additional risk estimates for all COVID-19 vaccines combined in comparison to influenza vaccines. However, some findings related to particular adverse reactions are worth mentioning here. On the one hand, the relative risk estimates for some adverse reactions were large, for instance pruritus, rashes, presyncope, myocardial infarction, myocarditis, pericarditis, pulmonary embolism, dysgeusia, cerebral haemorrhage, hemiparesis, paresthesia, seizures, renal pain, respiratory distress, acute respiratory failure, deep vein thrombosis, increased fibrin D dimer, menstrual disorder, thrombosis or vaginal haemorrhage, among several others. On the other hand, some serious reactions such as cerebral thrombosis and cerebral venous (sinus) thrombosis have been reported much more frequently after COVID-19 vaccination (combined mRNA and adenovirus-vectored vaccines) in comparison to influenza vaccines among adults. For instance, whereas 1229 and 157 cases of cerebral venous sinus thrombosis have been reported so far after COVID-19 vaccination, no cases have been reported for influenza vaccines in both EudraVigilance and VAERS, respectively (see Supplementary Material). In Table 4 the ten most frequent adverse reactions among reported deaths in EudraVigilance and VAERS are reported. In order to exclude unspecific reactions frequently mentioned in the death reports such as pyrexia, vomiting, or pain, the reactions in Table 4 focus on serious life-threatening conditions which might be related to the underlying causes of death. The comparison of the reported reactions across vaccine types suggests a substantial agreement between EudraVigilance and VAERS, with dyspnoea, respiratory arrest, pulmonary embolism, myocardial infarction, thrombosis, cerebral heamorrhages, and pneumonia being the adverse reactions most frequently mentioned in the death reports.

**TABLE 4:** The ten most frequent adverse reactions among reported deaths in EudraVigilance (EU) and VAERS (US).

**AstraZeneca (EU)**			
**Reaction**	**Cases**		
Pulmonary embolism	351		
Dyspnoea	339		
Thrombosis	219		
Cerebral haemorrhage	218		
Myocardial infarction	199		
Cerebral venous sinus thrombosis	128		
Cough	106		
Pneumonia	89		
Acute myocardial infarction	87		
Deep vein thrombosis	73		
**Moderna (EU)**		**Moderna (US)**	
**Reaction**	**Cases**	**Reaction**	**Cases**
Dyspnoea	342	Dyspnoea	433
Myocardial infarction	141	Pneumonia	147
Pneumonia	130	Cough	139
Pulmonary embolism	100	Myocardial infarction	117
Cardio-respiratory arrest	96	Hypoxia	101
Cough	88	Acute respiratory failure	94
Acute respiratory failure	74	Acute kidney injury	86
Respiratory failure	73	Hypotension	84
Respiratory arrest	69	Syncope	83
Hypotension	67	Cardio-respiratory arrest	77
**Janssen (EU)**		**Janssen (US)**	
**Reaction**	**Cases**	**Reaction**	**Cases**
Dyspnoea	91	Dyspnoea	116
Thrombosis	65	Cough	44
Cough	50	Acute respiratory failure	34
Myocardial infarction	47	Pulmonary embolism	34
Pulmonary embolism	43	Hypoxia	32
Cerebral haemorrhage	30	Thrombosis	22
Dizziness	21	Acute kidney injury	21
Cerebral venous sinus thrombosis	19	Hypotension	21
Deep vein thrombosis	19	Cerebral haemorrhage	19
Pneumonia	15	Pneumonia	19
**Pfizer (EU)**		**Pfizer (US)**	
**Reaction**	**Cases**	**Reaction**	**Cases**
Dyspnoea	519	Dyspnoea	513
Cardio-respiratory arrest	465	Cough	226
Myocardial infarction	375	Pneumonia	174
Pulmonary embolism	318	Hypoxia	158
Pneumonia	313	Acute respiratory failure	149
Cerebral haemorrhage	257	Acute kidney injury	127
Respiratory failure	190	Myocardial infarction	103
Acute myocardial infarction	159	Respiratory failure	95
Respiratory arrest	140	Syncope	82
Cerebral infarction	114	Cardio-respiratory arrest	75

*CTC categories excluded: Other reactions, allergic, constitutional, gastrointestinal, and pain reactions*.

## 4. Discussion

The findings of the present investigation indicate that the EudraVigilance and VAERS reports of the new COVID-19 vaccines are more frequently related to serious adverse outcomes, namely deaths, hospitalisations, and life-threatening reactions in comparison to the reports corresponding to influenza vaccines. The reported reactions associated with the new COVID-19 vaccines pertain more frequently allergic reactions, arrhythmia, cardiovascular events including myocardial infarction, cardiac or respiratory arrest, neurosensory disruptions, cerebrovascular accidents, haemorrhages, coagulopathy, pulmonary dysfunction, and thrombosis (see Supplementary Material for a detailed list of single reactions within CTC categories). The findings indicate a temporal relationship between vaccination and death events, since most reported serious adverse outcomes including death, hospitalisations, and life-threatening reactions are occurring within the first 7 days post-vaccination. Moreover, the relative risk estimates comparing the frequency of reported deaths between younger and older individuals suggest that the incidence of those acute life-threatening conditions are more frequent among individuals age 65 years and older. The reported symptoms of serious adverse reactions and the strength and direction of associations observed in the present study were consistent in the two databases included in the analyses, albeit the estimates obtained from the EudraVigilance reports are usually lower given the fact that the denominator of the influenza vaccine exposures considered only the population 65 years and older in the EU. Nonetheless, for the conclusions that can be drawn from the present investigation, the lower magnitude of the relative risk estimates in EudraVigilance should not pose any difficulties in interpreting the results, given the fact that the direction of associations are consistent in both reporting systems. For instance, there is a large excess risk of death, hospitalisation and life-threatening reports for all COVID-19 vaccines in comparison to the influenza vaccines (Table 1), and particularly large relative risks of thrombosis, coagulation and sexual organs reactions associated with COVID-19 vaccines (Tables 2b, 3b). Hence, it is clear that those reaction categories have a strong signal in both reporting systems, despite the differences in the exact numerical values of the effect size estimates.

When interpreting the numerical values of the estimates reported in this study, the readers should proceed with caution. It has to be emphasised that the numerical values of the relative risk estimates indicate a stronger or weaker signal which need to be interpreted taking into account risk assessment criteria, i.e., the clinical significance of the potential health hazards, the absolute risks of the particular adverse reaction and the risk levels tolerable for society (33, 34). For instance, a common reference value for acceptable lifetime cancer risk levels of exposure to carcinogens is around 4 × 10^−5^, i.e., about 10^−6^ per working year, assuming 40 years employment (34). In the context of the present investigation the interpretation of the numerical values can be illustrated as follows: The relative and absolute risks of reporting a spontaneous abortion in VAERS were 169.83 99% CI [71.65–402.55] and 0.423 per 100,000 exposures, whereas the corresponding estimates for vaccination site pain were 582.72 99% CI [369.33–919.42] and 5.165 per 100,000 exposures, respectively (see Supplementary Material). Thus, for the COVID-19 vaccination season 2021, the relative and absolute risks of reporting vaccination site pain in VAERS were about three to 11 times larger, respectively, than those of a spontaneous abortion. Nonetheless, the latter represents a less frequent, but more serious health hazard requiring further investigation than vaccination site pain which in most cases is not likely to result in serious or chronic health impairments. The assessment of the tolerable risks would depend on how societies weight the burden of the specific health hazards on population health.

From the perspective of drug safety, the risk estimates reported in this study can be interpreted as signals of new potentially causal associations or new aspects of known ones which may guide further verification actions in specific clinical studies (33). Although EMA and FDA have recognised so far about 30–40 adverse reactions following COVID-19 vaccination such as lymphadenopathy, allergic reactions, arthralgia, myalgia, myocarditis, and pericarditis (35, 36), the present investigation not only expands the scope on the potential health-adverse effects of the COVID-19 vaccines, but also calculates the signal strength of adverse reactions at the population level for seven major drug toxicity criteria (CTC categories) comprising 941 and 816 reported adverse reactions in EudraVigilance and VAERS, respectively (see Supplementary Material). The present findings indicate that there are multiple adverse reactions which have not been considered in the EMA and FDA product information sheets such as pulmonary, gastrointestinal, haemorrhage, neurological, sexual organs reactions, and thrombosis. In contrast to the routine reports issued by the health authorities in the EU and US, the present investigation provides also the relative risks for specific adverse reactions in comparison to the prophylactic influenza vaccines in use during 2020 and 2021. Thus, it is possible to interpret and evaluate the results in the context of relevant sources of vaccine-related toxicity, as recommended by the Council for International Organizations of Medical Sciences (CIOMS) (33). The adverse reactions with strong signals identified in the present study may represent the starting point for further studies using other sources of data such as death and hospitalisation registries in order to provide additional evidence of potentially causal associations. Furthermore, the results of the present study may be used to inform further signal prioritisation, triaging and evaluation of the public health impact of specific reactions (33).

The risk estimates of adverse reactions by vaccine type and CTC category were largest for the Pfizer-BioNTech vaccine in both EudraVigilance and VAERS, followed by the vaccines of AstraZeneca and Moderna. The COVID-19 vaccine of Janssen had usually lower absolute and relative risk estimates in both databases. Notwithstanding these differences at the level of CTC categories and the fact that the vaccines differ regarding the pharmaceutical technology, the ingredients and the number of doses required for full vaccination, the risk estimates of deaths, hospitalisations and life-threatening reactions were comparable across the mRNA and vectorised vaccines, implying a similar risk profile for both vaccine platforms. Even though more research is needed on the similarities and differences in the risk profile of the mRNA and vectorised vaccines, some of the pathophysiological pathways potentially leading to the observed risk profiles are discussed in the next section.

### 4.1. Potential Pathophysiological Mechanisms of Adverse Reactions

Since cancer immunotherapy constituted the major field of application of the nucleic-acid-based technology at the core of the COVID-19 vaccine platforms before 2019, the majority of previous findings on the pharmacokinetics of mRNA and chimeric virus vaccines were obtained from pre-clinical and clinical trials assessing their effects in the treatment of various cancer types such as melanoma, renal cancer, prostate cancer, leukaemia, or lung cancer (37, 38). On the contrary, previous research concerning the use of nucleic-acid-based technology in prophylactic vaccination, in particular for the mRNA platform, is much more limited (39, 40). Thus, by considering only available evidence from previous research on cancer immunotherapy, the spike S protein of SARS-CoV-2 and the pharmacokinetics of nanoparticles, the biological plausibility of the adverse reactions following COVID-19 vaccination can be summarised by the action of at least three major pathophysiological mechanisms. First, it is clear that the elicitation of strong immune responses must be a feature of both cancer immunotherapy and prophylactic vaccination, since their therapeutic effect is basically due to the building up of specific antigen-antibody production targeting the destruction of tumour cells in cancer immunotherapy and the induction of immunisation against viral infections in prophylactic vaccination, respectively. Hence, the nucleic-acid-based pharmaceutical technology on which the COVID-19 vaccines are based upon elicits potent immune responses via Toll-like receptos (TLR), interleukins (IL) IL-6, IL-12, interferon type 1 (IFN-1), tumour necrosis factor α (TNFα), pattern recognition receptors, dendritic cell maturation, induction of CD4+ and CD8+ T cell responses, among others (16, 17, 38, 41–44). At the same time, however, such potent immune reactions may also increase the risks of pathophysiological mechanisms related, for instance, to tissue and organ lesions and thromboembolic events (17, 45, 46). At least for the adenovirus-vector technology, results from clinical trials indicated that adenovirus proteins may elicit acute-phase immune responses involving the release of IL-6 and TNFα and activation of innate immunity cells such as mast cells and neutrophils (17, 41). In some instances, this may result in an increased likelihood of an acute shock-syndrome due to a cytokine cascade leading to disseminated intravascular coagulation, acute respiratory distress and multiorgan failure (45). In addition, by mechanisms which have not been fully explained so far, the pro-inflammatory environment related to the interactions between nucleic acids, TNFα, matured dendritic cells (DC) and the receptors TLR3 and TLR7 has been associated with disease progression of autoimmune diseases such as lupus erythematosus and rheumatoid arthritis (47–49).

Despite the advances made in the reduction of the pro-inflammatory risks of mRNA and vectorised pharmaceutical platforms [e.g., the use of pseudouridine in modified mRNA to reduce its adverse immunogenicity (50) or E2b^−^ modified adenovirus with reduced hepatotoxicity (51)], the induction of severe immune-induced reactions such as thrombocytopenia and human erythrocyte agglutination has been previously documented with adenovirus-vectorised therapies (52). Moreover, the present investigation suggests that all four nucleic-acid-based COVID-19 vaccines are associated with increased risks of thromboembolic events and, hence, they provide additional support for the results of a previous study with data from the Global Database for Individual Case Safety (VigiBase) in which endotheliopathy and coagulopathy had been observed also for all types of COVID-19 vaccines (53). From this perspective, the recently proposed “vaccine-induced immune thrombotic thrombocytopenia” (VITT) may be actually a severe manifestation in a continuum of vaccine-induced coagulopathy affecting to some degree vaccinated individuals (54, 55). In particular, the high frequency of reactions following COVID-19 vaccinations such as dyspnoea, pyrexia, cerebral haemorrhage, headache, headache, cardiac arrest and fatigue overlap with the typical signs and symptoms of acute pulmonary embolism (56), an adverse reaction which is more frequently reported in relation to COVID-19 vaccines than for influenza vaccines (see Supplementary Material). Moreover, the fact that the chances of reporting serious adverse reactions, especially deaths, largely increase with age (Table 1), suggest that some major vaccine-related risks may be associated with the age-dependent decay of haemodynamic and cardiovascular parameters such as co-morbid cardiovascular disease, endotheliopathy of (lower limb) veins, haemostasis and coagulation function which are directly related to thromboembolic risk (57, 58).

The second pathway is related to the known pathogenicity of the spike S of SARS-CoV-2 which has been involved in the endotheliopathy and coagulopathy observed in more severe forms of COVID-19: The spike S protein, expressed in both nucleic acid technologies of the COVID-19 vaccines reviewed here, is not only a potent activator of the alternative pathway of complement which may contribute to the endothelial damage observed in COVID-19 patients (59), but also an enhancer of platelet aggregation and thrombus formation (60). In addition, the spike subunit S1 can cross the blood-brain barrier and is taken up by the neural cells, the lung, liver, kidney and spleen (61). Hence, it is likely that the cleaved spike protein subunit in itself has the ability to cross other types of blood endothelial barriers surrounding immune privileged organs such as the spinal cord, ovaries, testes, pregnant uterus, placenta, and eyes (62), potentially inducing innate immune responses. Moreover, whereas adenovirus serotype 5 have been found to cross the blood brain barrier in the murine model (63), the nanolipid-complexed mRNA vaccine platform is optimised to diffuse across non-fenestrated endothelial blood barriers (64, 65) and, thus, due to the immune responses mentioned above, both vaccine platforms may induce in some cases a pro-inflammatory environment in the immune privileged organs. To some extent, this pathophysiological pathway involving transduction across blood barriers and subsequent immune response may partly explain some of the neurological and inflammatory reactions reported to VAERS and EudraVigilance affecting the central nervous system and the sexual organs (see Supplementary Material). Since previous pharmacokinetic results with male rats on the safety of mRNA encoding human-erythropoietin and complexed with lipid nanoparticles reported a more prolonged thromboplastin and prothrombin time in treated animals (66), it is possible that spike-induced erythrocyte agglutination and platelet activation may further contribute to increased thromboembolic event risk calculated for the mRNA COVID-19 vaccines. Finally, concerning the mRNA platform, a third pathway is related to the role of the lipid nanoparticles themselves used to complex the naked synthetic mRNA. Even though there have been advances to reduce the immunostimulation of lipid nanoparticles (e.g., by increasing the density of polyethylene glycol in the lipid nanoparticles (67)), they still may elicit pathogenic anaphylactoid reactions by complement activation (68–70) and enhanced platelet aggregation (71). These nanoparticle-related reactions may contribute to the pro-inflammatory host responses (66) and, consequently, to increased risks of thromboembolic or anaphylactoid outcomes. In particular, the complexed mRNA will tend to bio-accumulate in the adrenal and seminal vesicle wall, liver and spleen due to the normal lipid metabolism, bloodstream distribution and the permeability of the fenestrated endothelium to the lipid nanoparticles and, hence, these organs may become target organs of toxicity (72, 73). In fact, previous pharmacokinetic findings on the biodistribution of nanolipid, encapsulated nucleic-acid drugs revealed that the nanolipid vehicle prevents the nucleic-acid from being metabolised and, thus, blood and plasma concentrations of the nucleic-acid components are determined by the pharmacokinetics of the nanolipid vehicle (73).

The reactions commonly mentioned in the death reports such as pulmonary embolism, thrombosis, cerebral haemorrhage, myocardial infarction, cerebral venous sinus thrombosis (Table 4) are in agreement with the findings of previous autopsy studies which have identified several causal mechanisms linking COVID-19 vaccination and a lethal outcome. Of particular importance are strong immune-related life-threatening conditions involving antibody-mediated platelet activation in VITT cases (platelet factor 4) (74), neutrophil and histiocyte infiltrates in myocarditis (75), and reactive astrocytes, microglia, and foamy macrophaghes in cases of acute disseminated encephalomyelitis (neuro-inflammation) (76). Finally, the observed increased risks of death, hospitalisations, and life-threatening reactions among individuals age 65 years and older may be related to several age-dependent alterations of central biological functions and structures. In particular, with increasing ageing there seems to be an increased serum level of pro-inflammatory cytokines such as IL-6, IL-15, IL-8 (77) and multiple clotting factors including fibrinogen, factor VII, factor VIII, and von Willebrand factor (78). In addition, older individuals are affected by an increased risk of cardiovascular and cerebrovascular diseases due to the pathogenic alterations of the vasculature associated with atherosclerotic diseases, haemorrhages, aneurysms, vascular cognitive impairment, and microcirculation disruptions (79). Hence, given the potentially vaccine-induced pathophysiological mechanisms discussed above, these age-dependent alterations of the inflammatory response, vascular function and haemostasis may pre-dispose older individuals to an exacerbated inflammatory response, thrombus formation and endotheliopathy following COVID-19 vaccination which ultimately lead to the increased frequency of lethal outcomes, hospitalisations and life-threatening reactions among older individuals.

## 5. Strengths and Limitations

One of the major strengths of the present study is the availability of the number of individuals exposed to the new COVID-19 and influenza vaccines in the US and EU populations during 2020 and 2021 which allows a more accurate estimation of absolute risks of reporting adverse reactions. A major strength of the present investigation is the increased comparability of results in each reporting system, as the analyses were restricted to the last two reporting periods in the same surveillance systems, they involved large-scale prophylactic vaccines against respiratory viruses (SARS-CoV-2 and influenza) with a comparable number of exposures (451 million COVID-19 and 437 influenza vaccine exposures) and the populations are practically the same in 2020 and 2021 (i.e., almost the same individuals and demographic structure). On this account, varying sensitivity of the passive reporting systems can be ruled out as a major explanatory factor of the frequencies observed. The present study largely extends the information included in the reports of the health authorities insofar as the whole time series of adverse reactions reported to the pharmacovigilance surveillance systems of the EU and US are analysed and compared to each other according to established major toxicity criteria. To some extent, the present study is a replication study with two different reporting systems, vaccine types, populations, and health regulatory settings. Moreover, the risk estimates benefit from the fact that prior to 2020 the target populations were not exposed to SARS-CoV-2, the nucleic-acid and vectorised vaccines had never been used in the prophylactic vaccination of whole populations and there were no vaccines available against coronaviruses. This is an important strength of the present study in view of the rapidly increasing vaccine coverage rates against SARS-CoV-2 which will limit the availability of appropriate control groups made up of individuals without COVID-19 vaccine exposure. In addition, the present analyses are based on some of the largest datasets publicly available worldwide on vaccine-related adverse reactions containing approximately 7.8 million adverse reactions of 1.6 million individuals.

Nonetheless, there are at least four major limitations in the present study: (i) it has to be emphasised that the adverse reactions reports do not represent conclusive evidence of a causal association between vaccine exposure and adverse reaction, since they may also indicate correlations arising from the coincidental association of events following vaccination exposure or from unaccounted confounding factors such as concomitant medications or illnesses (23, 24), (ii) the collected data may also represent unverified reports of health events occurring after vaccination, (iii) they may affected by under- or over-reporting bias due to public awareness or saliency of certain reactions (21, 24), and (iv) the denominator used for the calculation of the influenza vaccination exposures in the EU is an under-estimate of the real number of exposures in the population 18 years and older. However, as the results of the present investigation suggest, the time series of reports are not correlated with the increasing vaccination coverage of persons aged 18 and older (Figure 1). Hence, public awareness or saliency of certain reactions does not seem to be an important source of bias concerning the frequency of reported adverse reactions. Even though it could be argued that increased awareness might explain the increased reporting rates concerning reactions such as injection site pain, myalgia, nausea, or vomiting, it is highly unlikely, on the contrary, that sudden serious medical conditions which require specific diagnostic procedures are merely due to increased awareness on COVID-19 vaccination: For instance, syncope, (acute) myocardial infarction, ischaemic stroke, pulmonary embolisms, pancreatitis, cerebral infarction, acute kidney injury, or deep vein thrombosis (see Supplementary Material). On the contrary, the time series of reported cases to EudraVigilance and VAERS seem to be decreasing over time, especially in the US. There may be several factors affecting the number of reports being recorded in EudraVigilance and VAERS such as delays of the database updates, increased costs of reporting adverse reactions due to the large number of persons having received at least 1 dose, changes in the reporting procedures or guidelines used in the health services institutions or unawareness of health professionals of potential adverse reactions related to the new COVID-19 vaccines. Further research is needed to assess why the reporting rates in VAERS and EudraVigilance differ and how the time series of reports and vaccine coverage are related to over- or under-estimation of particular adverse reactions. Finally, despite the fact that the number of influenza vaccination exposures in the EU is under-estimated, the relative risk estimates in EudraVigilance agree well in the direction and strength with the corresponding estimates in VAERS. As stated above, however, the exact numerical value of the relative risk estimates is less relevant in the context of risk assessment, signal prioritisation, tolerable risk levels and the clinical implications for the treatment of particular adverse reactions.

### 5.1. Potentially Causal Associations

Despite the limitations of passive reporting systems concerning causal associations, they may inform further clinical research investigating the extent to which the COVID vaccines can act as the main factor, or a secondary causal co-factor, increasing the probability of observing the adverse reactions identified in the passive reporting systems. In the EU, adverse events notified by healthcare professionals and consumers to the EudraVigilance report system are considered suspected adverse reactions implying that “a causal relationship between a medicinal product and an occurrence is suspected” (22, p. 6). According to the World Health Organization (WHO), the main criteria to be considered in the assessment of potentially causal associations are 1. the temporal relationship between vaccine exposure and reaction, 2. the strength of association suggesting a statistically significant increase of the conditional probability of observing the reaction after vaccine exposure, 3. the consistency of evidence across different studies or data sources, and 4. the biological plausibility between vaccine exposure and observed reaction (80). For the purposes of the present investigation, the temporal relationship and the consistency of evidence were evaluated by establishing the time of reaction onset and the comparison of the patterns of association found in the VAERS and EudraVigilance databases. The strength of associations was assessed by using the absolute risk estimates to calculate the relative risks of adverse reactions which may indicate potentially causal relationships. However, concerning the biological plausibility of the potentially causal associations, it is clear that only preliminary hypotheses can be formulated regarding the potential modes of action of the COVID-19 vaccines which may account for some of the observed adverse reactions. In the present investigation only such pathophysiological mechanisms were discussed which are supported by the findings of previous studies.

### 5.2. Future Research

Finally, the results of the present investigation may provide avenues for future clinical research on several areas. First, passive or spontaneous report systems suffer from serious under-estimation of adverse reactions. This is an important drawback, as the magnitude of under-reporting of non-serious and serious adverse reactions to spontaneous report systems has been estimated to lie in the range 91–99% and 92–98% in general practitioner and hospital settings, respectively (81). The reporting sensibility of adverse reactions such as rashes and thrombocytopenia in VAERS has been estimated to lie in the range 1% to 10%, as reported elsewhere (82). Although it cannot be completely ruled out that the reporting rates of COVID-19 vaccines may be to some extent higher than for the influenza vaccines, the major limitation of passive reporting systems is under-reporting rather than over-reporting: In general, the under-estimation of drug-related adverse reactions in spontaneous reporting systems has been calculated to be as high as 98%, so that single spontaneous reports “of a commonly occurring clinical event implies the existence of 50 more similar events in the total exposed patient population” (83, p. 343). Of course, future research should assess the magnitude of under-estimation and coverage of adverse reactions in EudraVigilance and VAERS in order to obtain more accurate risk estimates. Second, the investigation of the plausibility of different pathophysiological pathways needs to be further investigated in specific clinical studies. In particular, cohort studies collecting data on the haematological, immunological, neurological, and clinical profile of vaccinated individuals may provide a better assessment of the COVID-19 vaccine-related risk distribution at the population level. At the same time, additional autopsy studies may clarify the pathogenetic mechanisms potentially accounting for the reported death cases and/or life-threatening conditions. Despite the uncertainties concerning the exact causal relationships between the observed adverse reactions and COVID-19 vaccination, the present results may already help physicians develop prompt and adequate treatment protocols of individuals presenting serious medical conditions following COVID-19 vaccination. In face of the impending COVID-19 vaccine requirements and booster vaccination schedules implemented in several jurisdictions around the world, it is important to increase the research efforts in preventing lethal outcomes or life-threatening complications which may follow COVID-19 vaccination in some rare instances.

## 6. Conclusion

In the present investigation a higher risk of reporting serious adverse outcomes was observed for the COVID-19 vaccines in comparison to influenza vaccines deployed during 2020 and 2021. Individuals age 65 and older were associated with a higher frequency of death, hospitalisations, and life-threatening reactions than individuals age 18–64 years (relative risk estimates between 1.49 99% CI [1.44–1.55] and 8.61 99% CI [8.02–9.23]). The largest absolute risks related to COVID-19 vaccines corresponded to allergic, constitutional reactions, dermatological, gastrointestinal, neurological, and localised and non-localised pain. The largest relative risks between COVID-19 vs. influenza vaccines were observed for allergic reactions, arrhythmia, general cardiovascular events, coagulation, haemorrhages, constitutional, gastrointestinal, ocular, sexual organs reactions, and, in particular, thromboembolic events. Further clinical investigations are needed to identify both specific and common biological pathophysiological mechanisms across the different vaccine platforms, and to assess the relative safety between the different COVID-19 vaccines currently being deployed.

## Data Availability Statement

Data of Eudra Vigilance are publicly available as CSV files at https://www.adrreports.eu/ under the line listings view of the corresponding vaccine type. Data on vaccination coverage in the EU are available at https://www.ecdc.europa.eu/en/publications-data/data-covid-19-vaccination-eu-eea (download 26.10.2021). Data on population for the EU are available from Eurostat's database at https://ec.europa.eu/eurostat/web/main/data/database in the table population on 1 January by age, sex and educational attainment level (demo_pjanedu). Data on US vaccination coverage are available at https://data.cdc.gov/Vaccinations/COVID-19-Vaccinations-in-the-United-States-Jurisdi/unsk-b7fc (download 27.10.2021). Data of VAERS are publicly available as ZIP files for each reporting year at https://vaers.hhs.gov/data.html. Data on US annual resident population by age groups from 2010 to 2019 are available from the US Census Bureau at https://www.census.gov/en.html (table NC-EST2019).

## Author Contributions

DM conceived the research hypotheses, prepared and analysed the data and wrote all sections of the manuscript.

## Funding

We acknowledge support by Open Access Publishing Fund of University of Tübingen.

## Conflict of Interest

The author declares that the research was conducted in the absence of any commercial or financial relationships that could be construed as a potential conflict of interest.

## Publisher's Note

All claims expressed in this article are solely those of the authors and do not necessarily represent those of their affiliated organizations, or those of the publisher, the editors and the reviewers. Any product that may be evaluated in this article, or claim that may be made by its manufacturer, is not guaranteed or endorsed by the publisher.

## Abbreviations

CDC: Centres for Disease and Prevention
CTC: Common Toxicity Criteria
ECDC: European Centre for Disease and Prevention
EEA: European Economic Area
EMA: European Medicines Agency
EudraVigilance: European Database of Suspected Adverse Drug Reaction
MedDRA: Medical Dictionary for Regulatory Activities
FDA: US Food and Drug Administration
VITT: vaccine-induced immune thrombotic thrombocytopenia
VAERS: Vaccine Adverse Event Reporting System.
