# Supplementary material for: Frequency and Associations of Adverse Reactions of COVID-19 Vaccines Reported to Pharmacovigilance Systems in the European Union and the United States
Source: Front Public Health. 2022 Feb 3;9:756633. doi: 10.3389/fpubh.2021.756633 (PMC8850379; doi:10.3389/fpubh.2021.756633)
Supplement: Supplementary file 1 [file Data_Sheet_1.PDF]

Frequency and associations of adverse reactions of COVID-19 vaccines reported to pharmacovigilance systems in the European Union and the United States

Supplementary file

Table 1: Frequencies and proportions of serious adverse reactions in EudraVigilance and VAERS (2020-2021). Total number of reported reactions: 4,173,937 (1,096,569 persons) and 3,651,010 (534,332 persons), respectively. Reports of individuals age 18 years and older. LTR: life-threatening reaction.

**EudraVigilance**						
**Vaccine**	**Death**		**Hospitalisation**		**LTR**	
	**No**	**Yes**	**No**	**Yes**	**No**	**Yes**
COVID Astra-Zeneca	378919 (99.07)	3574 (0.93)	173485 (87.21)	25453 (12.79)	44933 (85.64)	7534 (14.36)
COVID-Janssen	30380 (97.66)	727 (2.34)	7373 (63.54)	4231 (36.46)	4598 (79.81)	1163 (20.19)
COVID Moderna	124165 (97.12)	3680 (2.88)	30699 (60.71)	19864 (39.29)	23933 (84.66)	4336 (15.34)
COVID Pfizer-BioNtech	466939 (98.33)	7929 (1.67)	135914 (75.79)	43420 (24.21)	59770 (86.63)	9221 (13.37)
Influenza	5261 (97.82)	117 (2.18)	1235 (66.01)	636 (33.99)	832 (87.03)	124 (12.97)
**Total**	1005664 (98.43)	16027 (1.57)	348706 (78.84)	93604 (21.16)	134066 (85.70)	22378 (14.30)

**VAERS**						
**Vaccine**	**Death**		**Hospitalisation**		**LTR**	
	**No**	**Yes**	**No**	**Yes**	**No**	**Yes**
COVID Janssen	44680 (98.60)	636 (1.40)	41646 (91.90)	3670 (8.10)	44208 (97.55)	1108 (2.45)
COVID Moderna	249173 (98.72)	3238 (1.28)	238783 (94.60)	13628 (5.40)	248876 (98.60)	3535 (1.40)
COVID Pfizer-BioNtech	223470 (98.59)	3204 (1.41)	210183 (92.72)	16491 (7.28)	222471 (98.15)	4203 (1.85)
Influenza	9895 (99.64)	36 (0.36)	9618 (96.85)	313 (3.15)	9852 (99.20)	79 (0.80)
**Total**	527218 (98.67)	7114 (1.33)	500230 (93.62)	34102 (6.38)	525407 (98.33)	8925 (1.67)

Table 2: Vaccine-related risk estimates of influenza (R_n) and COVID-19 vaccines (R_c) per 100,000 exposed individuals by Common Toxicity Criteria (CTC) and adverse reactions in the EudraVigilance database. Denominators of R_n and R_c: 7.76×10^7 65 years and older, and $246,534,547$ exposed individuals age 18 and older, respectively. Reactions without cases are left blank. Relative risks estimated only if at least 4 cases per reaction are available.

**CTC**	**Reaction**	**COVID cases**	**Influenza cases**	R_c	R_n	RR 99% CI
Allergic	Administration site pruritus	58	1	0.024		
Allergic	Allergic cough	36		0.015		
Allergic	Allergic respiratory disease	2	1			
Allergic	Allergic respiratory symptom	29		0.012		
Allergic	Anal pruritus	18		0.007		
Allergic	Application site pruritus	283	2	0.115		
Allergic	Brachioradial pruritus	2				
Allergic	Dermatitis allergic	925	16	0.375	0.021	17.82 [9.31–34.12]
Allergic	Dermatitis contact	86		0.035		
Allergic	Ear pruritus	114	2	0.046		
Allergic	Eye pruritus	1084	7	0.440	0.009	47.74 [17.98–126.78]
Allergic	Eyelids pruritus	79		0.032		
Allergic	Gingival pruritus	7		0.003		
Allergic	Hyperpyrexia	3699	14	1.500	0.018	81.45 [40.86–162.34]
Allergic	Implant site pruritus	1				
Allergic	Incision site pruritus	1				
Allergic	Infusion site pruritus	11		0.004		
Allergic	Injection site pruritus	9034	165	3.664	0.217	16.88 [13.79–20.66]
Allergic	Instillation site pruritus	1				
Allergic	Lip pruritus	194	2	0.079		
Allergic	Nasal pruritus	68		0.028		
Allergic	Oral pruritus	266	3	0.108		
Allergic	Parkinsonism hyperpyrexia syndrome	1				
Allergic	Pruritus	25186	270	10.216	0.355	28.76 [24.56–33.66]
Allergic	Pruritus allergic	33		0.013		
Allergic	Pruritus genital	41		0.017		
Allergic	Puerperal pyrexia	1				
Allergic	Puncture site pruritus	40		0.016		
Allergic	Pyrexia	269641	816	109.373	1.074	101.87 [93.07–111.49]
Allergic	Rash	26460	215	10.733	0.283	37.94 [31.80–45.26]

Continues on the next page ... . . .

CTC	Reaction	COVID	Influenza	R_c	R_n	RR 99% CI
Allergic	Rash erythematous	3922	37	1.591	0.049	32.68 [21.35–50.01]
Allergic	Rash follicular	6	1	0.002		
Allergic	Rash macular	2276	22	0.923	0.029	31.89 [18.37–55.38]
Allergic	Rash maculo-papular	583	13	0.236	0.017	13.82 [6.71–28.47]
Allergic	Rash maculovesicular	7		0.003		
Allergic	Rash morbilliform	157	2	0.064		
Allergic	Rash papular	1215	15	0.493	0.020	24.97 [12.79–48.76]
Allergic	Rash papulosquamous	3				
Allergic	Rash pruritic	5101	43	2.069	0.057	36.57 [24.65–54.25]
Allergic	Rash pustular	212	2	0.086		
Allergic	Rash rubelliform	7		0.003		
Allergic	Rash scarlatiniform	10		0.004		
Allergic	Rash vesicular	587	13	0.238	0.017	13.92 [6.76–28.66]
Allergic	Senile pruritus	1				
Allergic	Tongue pruritus	201	1	0.082		
Allergic	Urticaria	14455	205	5.863	0.270	21.74 [18.13–26.05]
Allergic	Vaccination site pruritus	3490	73	1.416	0.096	14.74 [10.87–19.99]
Allergic	Vulvovaginal pruritus	49		0.020		
Arrythmia	Arrhythmia	4313	16	1.749	0.021	83.10 [43.59–158.41]
Arrythmia	Arrhythmia neonatal	2				
Arrythmia	Arrhythmia supraventricular	57		0.023		
Arrythmia	Arrhythmogenic right ventricular dysplasia	2				
Arrythmia	Atrial tachycardia	50		0.020		
Arrythmia	Bradyarrhythmia	8		0.003		
Arrythmia	Bradycardia	1294		0.525		
Arrythmia	Bradycardia foetal	2				
Arrythmia	Bradycardia neonatal	1				
Arrythmia	Nodal arrhythmia	5		0.002		
Arrythmia	Pacemaker generated arrhythmia	2				
Arrythmia	Palpitations	19584	56	7.944	0.074	107.81 [76.37–152.18]
Arrythmia	Paroxysmal arrhythmia	8		0.003		
Arrythmia	Postural orthostatic tachycardia syndrome	156		0.063		
Arrythmia	Presyncope	6506	33	2.639	0.043	60.78 [38.77–95.27]
Arrythmia	Psychogenic pseudosyncope	1				
Arrythmia	Rebound tachycardia	1				
Arrythmia	Sinus arrhythmia	35		0.014		
Arrythmia	Sinus bradycardia	83		0.034		

Continues on the next page ... . . .

CTC	Reaction	COVID	Influenza	R_c	R_n	RR 99% CI
Arrythmia	Sinus tachycardia	540	5	0.219	0.007	33.29 [10.47–105.91]
Arrythmia	Supraventricular tachyarrhythmia	9		0.004		
Arrythmia	Supraventricular tachycardia	354	5	0.144	0.007	21.83 [6.84–69.63]
Arrythmia	Syncope	16798	76	6.814	0.100	68.14 [50.67–91.62]
Arrythmia	Tachyarrhythmia	131		0.053		
Arrythmia	Tachycardia	15674	43	6.358	0.057	112.37 [75.83–166.52]
Arrythmia	Tachycardia foetal	13		0.005		
Arrythmia	Tachycardia induced cardiomyopathy	3				
Arrythmia	Tachycardia paroxysmal	60		0.024		
Arrythmia	Ventricular arrhythmia	39		0.016		
Arrythmia	Ventricular tachyarrhythmia	6		0.002		
Arrythmia	Ventricular tachycardia	250	1	0.101		
Haematological	Granulocyte count	1				
Haematological	Granulocyte count decreased	1				
Haematological	Granulocyte count increased	7		0.003		
Haematological	Granulocyte percentage	4		0.002		
Haematological	Granulocytopenia	4		0.002		
Haematological	Granulocytosis	1				
Haematological	Lymphocele	6		0.002		
Haematological	Lymphocyte count	13		0.005		
Haematological	Lymphocyte count abnormal	8		0.003		
Haematological	Lymphocyte count decreased	107	1	0.043		
Haematological	Lymphocyte count increased	52		0.021		
Haematological	Lymphocyte count normal	4		0.002		
Haematological	Lymphocyte morphology abnormal	11		0.004		
Haematological	Lymphocyte percentage	5		0.002		
Haematological	Lymphocyte percentage abnormal	2				
Haematological	Lymphocyte percentage decreased	16	1	0.006		
Haematological	Lymphocyte percentage increased	4		0.002		
Haematological	Lymphocyte stimulation test positive	1				
Haematological	Lymphocytic infiltration	7	1	0.003		
Haematological	Lymphocytic leukaemia	1				
Haematological	Lymphocytosis	33	1	0.013		
Haematological	Lymphopenia	167		0.068		
Haematological	Neutrophil count	8		0.003		
Haematological	Neutrophil count abnormal	10		0.004		
Haematological	Neutrophil count decreased	74	1	0.030		

Continues on the next page ... . . .

CTC	Reaction	COVID	Influenza	R_c	R_n	RR 99% CI
Haematological	Neutrophil count increased	86	1	0.035		
Haematological	Neutrophil count normal	4		0.002		
Haematological	Neutrophil percentage	3				
Haematological	Neutrophil percentage abnormal	1				
Haematological	Neutrophil percentage decreased	3				
Haematological	Neutrophil percentage increased	15		0.006		
Haematological	Neutrophil toxic granulation present	4		0.002		
Haematological	Neutrophilia	61	1	0.025		
Haematological	Neutrophilic dermatosis	9		0.004		
Cardiovascular	Accelerated hypertension	18		0.007		
Cardiovascular	Acute left ventricular failure	33		0.013		
Cardiovascular	Acute myocardial infarction	1426	2	0.578		
Cardiovascular	Autoimmune myocarditis	3				
Cardiovascular	Autoimmune pericarditis	2				
Cardiovascular	Bacterial pericarditis	1				
Cardiovascular	Bone marrow ischaemia	1				
Cardiovascular	Brain stem ischaemia	8	1	0.003		
Cardiovascular	Cardio-respiratory arrest	827		0.335		
Cardiovascular	Cerebellar ischaemia	21		0.009		
Cardiovascular	Cerebral ischaemia	233		0.095		
Cardiovascular	Chronic left ventricular failure	11		0.004		
Cardiovascular	Cytomegalovirus pericarditis	1				
Cardiovascular	Diastolic hypertension	30		0.012		
Cardiovascular	Diastolic hypotension	6		0.002		
Cardiovascular	ECG signs of myocardial infarction	2				
Cardiovascular	ECG signs of myocardial ischaemia	2				
Cardiovascular	Eosinophilic myocarditis	3				
Cardiovascular	Gastrointestinal ischaemia	1				
Cardiovascular	Hepatic ischaemia	2				
Cardiovascular	Hypersensitivity myocarditis	1				
Cardiovascular	Hypertension	11987	44	4.862	0.058	83.98 [56.92–123.92]
Cardiovascular	Hypotension	5698	33	2.311	0.043	53.23 [33.95–83.45]
Cardiovascular	Immune-mediated myocarditis	3				
Cardiovascular	Infarction	171	1	0.069		
Cardiovascular	Infective pericardial effusion	1				
Cardiovascular	Injection site ischaemia	1				
Cardiovascular	Intestinal ischaemia	179		0.073		

Continues on the next page ... . . .

CTC	Reaction	COVID	Influenza	R_c	R_n	RR 99% CI
Cardiovascular	Ischaemia	183	3	0.074		
Cardiovascular	Ischaemic cardiomyopathy	19		0.008		
Cardiovascular	Ischaemic stroke	1802	6	0.731	0.008	92.58 [32.29–265.46]
Cardiovascular	Left ventricular dilatation	8		0.003		
Cardiovascular	Left ventricular dysfunction	93		0.038		
Cardiovascular	Left ventricular end-diastolic pressure	1				
Cardiovascular	Left ventricular end-diastolic pressure increased	2				
Cardiovascular	Left ventricular enlargement	7		0.003		
Cardiovascular	Left ventricular failure	70		0.028		
Cardiovascular	Left ventricular hypertrophy	48		0.019		
Cardiovascular	Myocardial infarction	3171	5	1.286	0.007	195.51 [61.73–619.21]
Cardiovascular	Myocardial ischaemia	199	3	0.081		
Cardiovascular	Myocarditis	5356	15	2.173	0.020	110.07 [56.55–214.25]
Cardiovascular	Myocarditis infectious	3				
Cardiovascular	Myocarditis septic	2				
Cardiovascular	Orthostatic hypertension	11		0.004		
Cardiovascular	Orthostatic hypotension	298	2	0.121		
Cardiovascular	Pericardial effusion	887	4	0.360	0.005	68.36 [18.80–248.54]
Cardiovascular	Pericarditis	4275	14	1.734	0.018	94.13 [47.24–187.59]
Cardiovascular	Pericarditis constrictive	13		0.005		
Cardiovascular	Pericarditis infective	8		0.003		
Cardiovascular	Peripheral ischaemia	212	1	0.086		
Cardiovascular	Phlebitis	569	2	0.231		
Cardiovascular	Phlebitis deep	18		0.007		
Cardiovascular	Phlebitis superficial	131	1	0.053		
Cardiovascular	Pleuropericarditis	52		0.021		
Cardiovascular	Pulmonary embolism	11653	15	4.727	0.020	239.49 [123.10–465.92]
Cardiovascular	Purulent pericarditis	1				
Cardiovascular	Renal ischaemia	11		0.004		
Cardiovascular	Retinal ischaemia	14		0.006		
Cardiovascular	Septic pulmonary embolism	2				
Cardiovascular	Silent myocardial infarction	3				
Cardiovascular	Spinal cord ischaemia	15		0.006		
Cardiovascular	Spleen ischaemia	2				
Cardiovascular	Subendocardial ischaemia	1				
Cardiovascular	Sudden cardiac death	109		0.044		

Continues on the next page ... . . .

CTC	Reaction	COVID	Influenza	R_c	R_n	RR 99% CI
Cardiovascular	Systolic hypertension	31		0.013		
Cardiovascular	Transient ischaemic attack	2369	3	0.961		
Cardiovascular	Troponin	35		0.014		
Cardiovascular	Troponin abnormal	24		0.010		
Cardiovascular	Troponin decreased	1				
Cardiovascular	Troponin I	8		0.003		
Cardiovascular	Troponin I abnormal	1				
Cardiovascular	Troponin I decreased	2				
Cardiovascular	Troponin I increased	49		0.020		
Cardiovascular	Troponin I normal	4		0.002		
Cardiovascular	Troponin increased	491	3	0.199		
Cardiovascular	Troponin normal	20		0.008		
Cardiovascular	Troponin T	1				
Cardiovascular	Troponin T increased	57		0.023		
Cardiovascular	Vaccination site ischaemia	1				
Cardiovascular	Vestibular ischaemia	1				
Cardiovascular	Viral myocarditis	22		0.009		
Cardiovascular	Viral pericarditis	30		0.012		
Coagulation	Activated partial thromboplastin time	18		0.007		
Coagulation	Activated partial thromboplastin time prolonged	204		0.083		
Coagulation	Activated partial thromboplastin time ratio increased	1				
Coagulation	Activated partial thromboplastin time shortened	45		0.018		
Coagulation	Coagulation factor decreased	1				
Coagulation	Coagulation factor increased	8		0.003		
Coagulation	Coagulation factor VIII level decreased	4		0.002		
Coagulation	Coagulation factor VIII level increased	4		0.002		
Coagulation	Coagulation test abnormal	21		0.009		
Coagulation	Coagulation time abnormal	2				
Coagulation	Coagulation time prolonged	29		0.012		
Coagulation	Coagulopathy	639		0.259		
Coagulation	Congenital hypercoagulation	1				
Coagulation	Disseminated intravascular coagulation	228		0.092		
Coagulation	Disseminated intravascular coagulation in newborn	1				

Continues on the next page ... . . .

CTC	Reaction	COVID	Influenza	R_c	R_n	RR 99% CI
Coagulation	Fibrin D dimer	93		0.038		
Coagulation	Fibrin D dimer decreased	15		0.006		
Coagulation	Fibrin D dimer increased	2001	2	0.812		
Coagulation	Fibrin degradation products increased	8		0.003		
Coagulation	Hypercoagulation	69		0.028		
Coagulation	Immune thrombocytopenia	1549	8	0.628	0.011	59.69 [23.95–148.74]
Coagulation	ISTH score for disseminated intravascular coagulation	1				
Coagulation	Neonatal alloimmune thrombocytopenia	6		0.002		
Coagulation	Platelet count	30		0.012		
Coagulation	Platelet count abnormal	37		0.015		
Coagulation	Platelet count decreased	1436	3	0.582		
Coagulation	Platelet count increased	175		0.071		
Coagulation	Platelet disorder	58		0.024		
Coagulation	Prothrombin level	1				
Coagulation	Prothrombin level decreased	3				
Coagulation	Prothrombin level increased	11		0.004		
Coagulation	Prothrombin time	22		0.009		
Coagulation	Prothrombin time prolonged	40		0.016		
Coagulation	Prothrombin time ratio increased	9		0.004		
Coagulation	Prothrombin time shortened	18		0.007		
Constitutional	Ascites	128		0.052		
Constitutional	Body temperature abnormal	302	3	0.122		
Constitutional	Body temperature increased	17538	97	7.114	0.128	55.74 [42.88–72.45]
Constitutional	Chills	185416	335	75.209	0.441	170.62 [148.21–196.43]
Constitutional	Fatigue	236016	550	95.733	0.724	132.29 [118.51–147.66]
Constitutional	Fatigue management	1				
Constitutional	Lethargy	7573	19	3.072	0.025	122.87 [68.00–222.03]
Constitutional	Malaise	137870	381	55.923	0.501	111.55 [97.74–127.31]
Constitutional	Mental fatigue	747		0.303		
Constitutional	Muscle fatigue	1609	7	0.653	0.009	70.86 [26.71–187.99]
Constitutional	Sweating fever	820		0.333		
Dermatological	Dermatitis	599	12	0.243	0.016	15.39 [7.26–32.61]
Dermatological	Erythema	16821	208	6.823	0.274	24.93 [20.83–29.84]
Dermatological	Erythema ab igne	1				
Dermatological	Erythema annulare	22	1	0.009		
Dermatological	Erythema dyschromicum perstans	1				

Continues on the next page ... . . .

CTC	Reaction	COVID	Influenza	R_c	R_n	RR 99% CI
Dermatological	Erythema elevatum diutinum	4		0.002		
Dermatological	Erythema induratum	19	1	0.008		
Dermatological	Erythema infectiosum	1				
Dermatological	Erythema migrans	20		0.008		
Dermatological	Erythema multiforme	521	3	0.211		
Dermatological	Erythema nodosum	229	2	0.093		
Dermatological	Erythema of eyelid	136	1	0.055		
Dermatological	Erythematotelangiectatic rosacea	3				
Dermatological	Flushing	3650	32	1.481	0.042	35.16 [22.26–55.55]
Dermatological	Injection site erythema	23003	507	9.331	0.667	13.99 [12.46–15.70]
Dermatological	Injection site inflammation	25438	486	10.318	0.639	16.14 [14.34–18.16]
Dermatological	Injection site reaction	5857	37	2.376	0.049	48.80 [31.91–74.63]
Dermatological	Injection site swelling	25502	445	10.344	0.586	17.67 [15.62–19.98]
Dermatological	Injection site warmth	21300	231	8.640	0.304	28.43 [23.97–33.71]
Gastrointestinal	Allergic stomatitis	1				
Gastrointestinal	Dehydration	1948	5	0.790	0.007	120.10 [37.90–380.61]
Gastrointestinal	Diarrhoea	37728	206	15.303	0.271	56.46 [47.16–67.59]
Gastrointestinal	Duodenal ulcer	11		0.004		
Gastrointestinal	Dysgeusia	5328	24	2.161	0.032	68.44 [40.40–115.92]
Gastrointestinal	Dyspepsia	2286	5	0.927	0.007	140.94 [44.48–446.55]
Gastrointestinal	Dysphagia	3175	28	1.288	0.037	34.96 [21.44–57.00]
Gastrointestinal	Gastric ulcer	76		0.031		
Gastrointestinal	Gastritis	399	2	0.162		
Gastrointestinal	Ileus	56		0.023		
Gastrointestinal	Malignant dysphagia	1				
Gastrointestinal	Nausea	147472	386	59.818	0.508	117.78 [103.29–134.30]
Gastrointestinal	Necrotising ulcerative gingivostomatitis	2				
Gastrointestinal	Oesophagitis	55		0.022		
Gastrointestinal	Oesophagitis ulcerative	2				
Gastrointestinal	Pancreatitis	323	3	0.131		
Gastrointestinal	Stomatitis	521	2	0.211		
Gastrointestinal	Stomatitis haemorrhagic	1				
Gastrointestinal	Stomatitis necrotising	1				
Gastrointestinal	Vomiting	40915	187	16.596	0.246	67.45 [55.85–81.46]
Haemorraghe	Adrenal haemorrhage	31		0.013		
Haemorraghe	Anal haemorrhage	100	1	0.041		
Haemorraghe	Arterial haemorrhage	8		0.003		

Continues on the next page ... . . .

CTC	Reaction	COVID	Influenza	R_c	R_n	RR 99% CI
Haemorraghe	Basal ganglia haematoma	1				
Haemorraghe	Basal ganglia haemorrhage	28		0.011		
Haemorraghe	Basal ganglia infarction	25		0.010		
Haemorraghe	Basal ganglia stroke	24		0.010		
Haemorraghe	Brain stem haematoma	1				
Haemorraghe	Brain stem haemorrhage	51		0.021		
Haemorraghe	Brain stem infarction	117	1	0.047		
Haemorraghe	Brain stem stroke	42		0.017		
Haemorraghe	Bullous haemorrhagic dermatosis	10		0.004		
Haemorraghe	Diarrhoea haemorrhagic	298	1	0.121		
Haemorraghe	Diverticulitis intestinal haemorrhagic	2				
Haemorraghe	Diverticulum intestinal haemorrhagic	9		0.004		
Haemorraghe	Duodenal ulcer haemorrhage	13		0.005		
Haemorraghe	Embolic stroke	160		0.065		
Haemorraghe	Gastric haemorrhage	61	1	0.025		
Haemorraghe	Gastric ulcer haemorrhage	14		0.006		
Haemorraghe	Gastrointestinal haemorrhage	289		0.117		
Haemorraghe	Haemorrhage	3670	2	1.489		
Haemorraghe	Haemorrhage subcutaneous	274	1	0.111		
Haemorraghe	Haemorrhage subepidermal	3				
Haemorraghe	Haemorrhage urinary tract	189	1	0.077		
Haemorraghe	Haemorrhoid infection	1				
Haemorraghe	Haemorrhoid operation	2				
Haemorraghe	Haemorrhoidal haemorrhage	59	1	0.024		
Haemorraghe	Haemorrhoids	286	1	0.116		
Haemorraghe	Haemorrhoids thrombosed	149		0.060		
Haemorraghe	Hepatic haemorrhage	8		0.003		
Haemorraghe	Intestinal haemorrhage	69	2	0.028		
Haemorraghe	Intra-abdominal haemorrhage	12		0.005		
Haemorraghe	Large intestinal haemorrhage	11		0.004		
Haemorraghe	Large intestinal ulcer haemorrhage	1				
Haemorraghe	Lower gastrointestinal haemorrhage	8		0.003		
Haemorraghe	Mouth haemorrhage	296		0.120		
Haemorraghe	Mucosal haemorrhage	41	1	0.017		
Haemorraghe	Muscle haemorrhage	31		0.013		
Haemorraghe	Oesophageal ulcer haemorrhage	1				
Haemorraghe	Pelvic haemorrhage	51		0.021		

Continues on the next page ... . . .

CTC	Reaction	COVID	Influenza	R_c	R_n	RR 99% CI
Haemorraghe	Peptic ulcer haemorrhage	22		0.009		
Haemorraghe	Pericardial haemorrhage	33		0.013		
Haemorraghe	Petechiae	2697	9	1.094	0.012	92.38 [39.09–218.32]
Haemorraghe	Pharyngeal haemorrhage	23		0.009		
Haemorraghe	Pulmonary haemorrhage	60		0.024		
Haemorraghe	Rectal haemorrhage	628	3	0.255		
Haemorraghe	Renal haemorrhage	23		0.009		
Haemorraghe	Respiratory tract haemorrhage	15	1	0.006		
Haemorraghe	Shock haemorrhagic	34		0.014		
Haemorraghe	Skin haemorrhage	413		0.168		
Haemorraghe	Skin ulcer haemorrhage	1				
Haemorraghe	Small intestinal ulcer haemorrhage	1				
Haemorraghe	Spinal subarachnoid haemorrhage	1				
Haemorraghe	Spinal subdural haematoma	1				
Haemorraghe	Spinal subdural haemorrhage	1				
Haemorraghe	Spontaneous haemorrhage	22		0.009		
Haemorraghe	Subarachnoid haematoma	5		0.002		
Haemorraghe	Subarachnoid haemorrhage	584	3	0.237		
Haemorraghe	Subdural haematoma	193		0.078		
Haemorraghe	Subdural haematoma evacuation	2				
Haemorraghe	Subdural haemorrhage	46		0.019		
Haemorraghe	Subdural hygroma	2				
Haemorraghe	Tongue haemorrhage	21		0.009		
Haemorraghe	Ulcer haemorrhage	24		0.010		
Haemorraghe	Upper gastrointestinal haemorrhage	53		0.021		
Haemorraghe	Urethral haemorrhage	8		0.003		
Haemorraghe	Urinary bladder haemorrhage	43		0.017		
Haemorraghe	Vaccination site haemorrhage	227	19	0.092	0.025	3.68 [1.99–6.81]
Haemorraghe	Venous haemorrhage	21		0.009		
Haemorraghe	Wound haemorrhage	53	2	0.021		
Neurological	Acquired epileptic aphasia	1				
Neurological	Action tremor	9		0.004		
Neurological	Acute motor axonal neuropathy	14	1	0.006		
Neurological	Acute motor-sensory axonal neuropathy	11		0.004		
Neurological	Acute polyneuropathy	64	5	0.026	0.007	3.95 [1.19–13.05]
Neurological	Administration site dysaesthesia	3				
Neurological	Administration site hypoaesthesia	4		0.002		

Continues on the next page ... . . .

CTC	Reaction	COVID	Influenza	R_c	R_n	RR 99% CI
Neurological	Administration site paraesthesia	16		0.006		
Neurological	Anaesthesia	252		0.102		
Neurological	Anal hypoaesthesia	8		0.003		
Neurological	Anal paraesthesia	1				
Neurological	Aphasia	1912	12	0.776	0.016	49.12 [23.30–103.56]
Neurological	Application site dysaesthesia	3				
Neurological	Application site hypoaesthesia	49	1	0.020		
Neurological	Application site paraesthesia	31		0.013		
Neurological	Ataxia	309	6	0.125	0.008	15.88 [5.49–45.90]
Neurological	Autoimmune neuropathy	12	1	0.005		
Neurological	Autonomic neuropathy	21		0.009		
Neurological	Axonal and demyelinating polyneuropathy	14		0.006		
Neurological	Axonal neuropathy	22		0.009		
Neurological	Cerebral amyloid angiopathy	11		0.004		
Neurological	Cerebral arteriosclerosis	12		0.005		
Neurological	Cerebral arteritis	2				
Neurological	Cerebral artery embolism	89		0.036		
Neurological	Cerebral artery occlusion	103		0.042		
Neurological	Cerebral artery perforation	2				
Neurological	Cerebral artery stenosis	19		0.008		
Neurological	Cerebral artery stent insertion	1				
Neurological	Cerebral ataxia	1				
Neurological	Cerebral atrophy	32		0.013		
Neurological	Cerebral calcification	6		0.002		
Neurological	Cerebral capillary telangiectasia	1				
Neurological	Cerebral cavernous malformation	5		0.002		
Neurological	Cerebral circulatory failure	2				
Neurological	Cerebral congestion	15		0.006		
Neurological	Cerebral cyst	3				
Neurological	Cerebral disorder	84		0.034		
Neurological	Cerebral endovascular aneurysm repair	3				
Neurological	Cerebral haemangioma	3				
Neurological	Cerebral haematoma	106		0.043		
Neurological	Cerebral haemorrhage	1605	2	0.651		
Neurological	Cerebral haemorrhage foetal	1				
Neurological	Cerebral haemorrhage neonatal	1				
Neurological	Cerebral haemosiderin deposition	2				

Continues on the next page ... . . .

CTC	Reaction	COVID	Influenza	R_c	R_n	RR 99% CI
Neurological	Cerebral hyperperfusion syndrome	1				
Neurological	Cerebral hypoperfusion	4		0.002		
Neurological	Cerebral infarction	1940	5	0.787	0.007	119.61 [37.74–379.05]
Neurological	Cerebral infarction foetal	2				
Neurological	Cerebral mass effect	52		0.021		
Neurological	Cerebral microangiopathy	10		0.004		
Neurological	Cerebral microembolism	3				
Neurological	Cerebral microhaemorrhage	17		0.007		
Neurological	Cerebral microinfarction	6		0.002		
Neurological	Cerebral palsy	18	1	0.007		
Neurological	Cerebral reperfusion injury	1				
Neurological	Cerebral revascularisation	2				
Neurological	Cerebral salt-wasting syndrome	1				
Neurological	Cerebral small vessel ischaemic disease	59		0.024		
Neurological	Cerebral vascular occlusion	8		0.003		
Neurological	Cerebral vasoconstriction	10		0.004		
Neurological	Cerebral ventricle collapse	1				
Neurological	Cerebral ventricle dilatation	5		0.002		
Neurological	Cerebral ventricular rupture	14		0.006		
Neurological	Cervical spinal cord paralysis	1				
Neurological	Cervicogenic vertigo	3				
Neurological	Chronic inflammatory demyelinating polyradiculoneuropathy	67		0.027		
Neurological	Cold dysaesthesia	1				
Neurological	Confusional arousal	4		0.002		
Neurological	Cranial nerve paralysis	24		0.010		
Neurological	Delusion	196	3	0.080		
Neurological	Demyelinating polyneuropathy	69		0.028		
Neurological	Dental dysaesthesia	4		0.002		
Neurological	Dental paraesthesia	34		0.014		
Neurological	Diabetic mononeuropathy	1				
Neurological	Diabetic neuropathy	6		0.002		
Neurological	Diaphragmatic paralysis	4		0.002		
Neurological	Diplegia	257	7	0.104	0.009	11.32 [4.22–30.36]
Neurological	Dizziness	79928	275	32.421	0.362	89.60 [76.69–104.68]
Neurological	Dizziness exertional	203		0.082		
Neurological	Dizziness postural	2840	5	1.152	0.007	175.10 [55.28–554.64]

Continues on the next page ... . . .

CTC	Reaction	COVID	Influenza	R_c	R_n	RR 99% CI
Neurological	Dysaesthesia	796	8	0.323	0.011	30.67 [12.28–76.60]
Neurological	Dysaesthesia pharynx	3				
Neurological	Dystonic tremor	4		0.002		
Neurological	Enteric neuropathy	1				
Neurological	Essential tremor	15		0.006		
Neurological	Extraocular muscle paresis	58		0.024		
Neurological	Eye paraesthesia	45		0.018		
Neurological	Facial paralysis	5889	30	2.389	0.039	60.51 [37.77–96.97]
Neurological	Facial paresis	1612	13	0.654	0.017	38.23 [18.66–78.32]
Neurological	Familial periodic paralysis	5		0.002		
Neurological	Familial tremor	1				
Neurological	Genital dysaesthesia	1				
Neurological	Genital hypoaesthesia	5		0.002		
Neurological	Genital paraesthesia	6		0.002		
Neurological	Guillain-Barre syndrome	2586	109	1.049	0.143	7.31 [5.69–9.41]
Neurological	Hemidysaesthesia	48		0.019		
Neurological	Hemiparaesthesia	380	1	0.154		
Neurological	Hemiparesis	1584	10	0.643	0.013	48.83 [21.57–110.55]
Neurological	Hemiplegia	742	2	0.301		
Neurological	Hereditary neuropathy with liability to pressure palsies	4		0.002		
Neurological	Hoover's sign of leg paresis	2				
Neurological	Hypoaesthesia	21169	113	8.587	0.149	57.75 [45.29–73.63]
Neurological	Hypoaesthesia eye	81		0.033		
Neurological	Hypoaesthesia oral	2839	20	1.152	0.026	43.76 [24.55–78.00]
Neurological	Hypoaesthesia teeth	15		0.006		
Neurological	Hypoglossal nerve paralysis	5		0.002		
Neurological	Hypoglossal nerve paresis	1				
Neurological	IIIrd nerve paralysis	93		0.038		
Neurological	IIIrd nerve paresis	22		0.009		
Neurological	Immune-mediated neuropathy	6		0.002		
Neurological	Implant site hypoaesthesia	2				
Neurological	Implant site paraesthesia	3				
Neurological	Infusion site hypoaesthesia	4		0.002		
Neurological	Initial insomnia	367	2	0.149		
Neurological	Injection site dysaesthesia	14		0.006		
Neurological	Injection site hypoaesthesia	291	4	0.118	0.005	22.43 [6.13–82.02]

Continues on the next page ... . . .

CTC	Reaction	COVID	Influenza	R_c	R_n	RR 99% CI
Neurological	Injection site paraesthesia	358	3	0.145		
Neurological	Insomnia	13288	45	5.390	0.059	91.03 [61.96–133.73]
Neurological	Intention tremor	11		0.004		
Neurological	Intranasal hypoaesthesia	9		0.004		
Neurological	Intranasal paraesthesia	5		0.002		
Neurological	Ischaemic neuropathy	5		0.002		
Neurological	IVth nerve paralysis	30		0.012		
Neurological	IVth nerve paresis	5		0.002		
Neurological	Laryngeal tremor	1				
Neurological	Medical device site hypoaesthesia	1				
Neurological	Memory impairment	1996	12	0.810	0.016	51.28 [24.32–108.10]
Neurological	Middle insomnia	337	5	0.137	0.007	20.78 [6.51–66.31]
Neurological	Mononeuropathy	22		0.009		
Neurological	Mononeuropathy multiplex	10		0.004		
Neurological	Monoparesis	486	2	0.197		
Neurological	Monoplegia	838	9	0.340	0.012	28.70 [12.11–68.05]
Neurological	Multifocal motor neuropathy	3				
Neurological	Neuronal neuropathy	2				
Neurological	Neuropathy peripheral	1907	12	0.774	0.016	48.99 [23.24–103.29]
Neurological	Nystagmus	227	2	0.092		
Neurological	Oculofacial paralysis	6		0.002		
Neurological	Ophthalmoplegia	72	1	0.029		
Neurological	Optic ischaemic neuropathy	136		0.055		
Neurological	Optic neuropathy	38	2	0.015		
Neurological	Oral dysaesthesia	61		0.025		
Neurological	Orthostatic tremor	1				
Neurological	Palmar-plantar erythrodysaesthesia syndrome	26		0.011		
Neurological	Paradoxical insomnia	1				
Neurological	Paraesthesia	35328	156	14.330	0.205	69.81 [56.78–85.84]
Neurological	Paraesthesia ear	29		0.012		
Neurological	Paraesthesia mucosal	17		0.007		
Neurological	Paraesthesia oral	4805	36	1.949	0.047	41.15 [26.74–63.31]
Neurological	Paralysis	1570	10	0.637	0.013	48.40 [21.38–109.58]
Neurological	Paralysis recurrent laryngeal nerve	9		0.004		
Neurological	Paraparesis	90	3	0.037		
Neurological	Paraplegia	64	3	0.026		

Continues on the next page ... . . .

CTC	Reaction	COVID	Influenza	R_c	R_n	RR 99% CI
Neurological	Paresis	304	1	0.123		
Neurological	Paresis cranial nerve	13		0.005		
Neurological	Parkinsonian rest tremor	2				
Neurological	Peripheral motor neuropathy	18		0.007		
Neurological	Peripheral nerve paresis	15		0.006		
Neurological	Peripheral paralysis	34	2	0.014		
Neurological	Peripheral sensorimotor neuropathy	15	1	0.006		
Neurological	Peripheral sensory neuropathy	86	1	0.035		
Neurological	Persistent postural-perceptual dizziness	46		0.019		
Neurological	Pharyngeal hypoaesthesia	187	3	0.076		
Neurological	Pharyngeal paraesthesia	373	6	0.151	0.008	19.16 [6.64–55.32]
Neurological	Phobic postural vertigo	10		0.004		
Neurological	Phrenic nerve paralysis	3				
Neurological	Polyneuropathy	325	11	0.132	0.014	9.11 [4.13–20.06]
Neurological	Polyneuropathy chronic	3				
Neurological	Polyneuropathy idiopathic progressive	3				
Neurological	Postictal paralysis	12		0.005		
Neurological	Postural tremor	6		0.002		
Neurological	Procedural dizziness	38		0.015		
Neurological	Pseudoparalysis	2				
Neurological	Psychogenic tremor	3				
Neurological	Putamen haemorrhage	30		0.012		
Neurological	Quadriparesis	66	2	0.027		
Neurological	Quadriplegia	49	2	0.020		
Neurological	Respiratory paralysis	3	1			
Neurological	Resting tremor	30		0.012		
Neurological	Sciatic nerve neuropathy	8		0.003		
Neurological	Seizure	6500	23	2.637	0.030	87.12 [50.87–149.21]
Neurological	Seizure anoxic	11		0.004		
Neurological	Seizure cluster	25		0.010		
Neurological	Seizure like phenomena	147	5	0.060	0.007	9.06 [2.81–29.24]
Neurological	Seizure prophylaxis	1				
Neurological	Sensory neuropathy hereditary	1				
Neurological	Sleep disorder due to general medical condition, insomnia type	56		0.023		
Neurological	Sleep paralysis	63	1	0.026		
Neurological	Small fibre neuropathy	42		0.017		

Continues on the next page ... . . .

CTC	Reaction	COVID	Influenza	R_c	R_n	RR 99% CI
Neurological	Subacute inflammatory demyelinating polyneuropathy	14	1	0.006		
Neurological	Terminal insomnia	52		0.021		
Neurological	Thermohypoaesthesia	10		0.004		
Neurological	Tongue paralysis	62		0.025		
Neurological	Toxic neuropathy	1				
Neurological	Transient aphasia	25		0.010		
Neurological	Tremor	18937	66	7.681	0.087	88.45 [64.38–121.52]
Neurological	Trigeminal nerve paresis	11		0.004		
Neurological	Vaccination site dysaesthesia	52	1	0.021		
Neurological	Vaccination site hypoaesthesia	229	3	0.093		
Neurological	Vaccination site paraesthesia	468	8	0.190	0.011	18.03 [7.20–45.18]
Neurological	Vagus nerve paralysis	3				
Neurological	Vasoplegia syndrome	8		0.003		
Neurological	Vertigo	12973	66	5.262	0.087	60.59 [44.09–83.27]
Neurological	Vertigo CNS origin	8		0.003		
Neurological	Vertigo labyrinthine	24		0.010		
Neurological	Vertigo positional	522	1	0.212		
Neurological	VIth nerve paralysis	121	2	0.049		
Neurological	VIth nerve paresis	9		0.004		
Neurological	Vocal cord paralysis	46		0.019		
Neurological	Vocal cord paresis	8		0.003		
Ocular	Central vision loss	11		0.004		
Ocular	Colour vision tests abnormal	1				
Ocular	Computer vision syndrome	1				
Ocular	Diplopia	1761	12	0.714	0.016	45.24 [21.45–95.40]
Ocular	Dry eye	843	4	0.342	0.005	64.97 [17.87–236.25]
Ocular	Eye abscess	6		0.002		
Ocular	Eye allergy	50	2	0.020		
Ocular	Eye colour change	36		0.015		
Ocular	Eye complication associated with device	1				
Ocular	Eye contusion	106		0.043		
Ocular	Eye degenerative disorder	1				
Ocular	Eye discharge	119	2	0.048		
Ocular	Eye disorder	504	4	0.204	0.005	38.84 [10.66–141.53]
Ocular	Eye drop instillation	1				
Ocular	Eye haematoma	85		0.034		

Continues on the next page ... . . .

CTC	Reaction	COVID	Influenza	R_c	R_n	RR 99% CI
Ocular	Eye haemorrhage	739		0.300		
Ocular	Eye infarction	42		0.017		
Ocular	Eye infection	144		0.058		
Ocular	Eye infection bacterial	9		0.004		
Ocular	Eye infection toxoplasmal	4		0.002		
Ocular	Eye infection viral	6		0.002		
Ocular	Eye inflammation	378	5	0.153	0.007	23.31 [7.31–74.31]
Ocular	Eye injury	135		0.055		
Ocular	Eye irrigation	6		0.002		
Ocular	Eye irritation	1297	7	0.526	0.009	57.12 [21.52–151.61]
Ocular	Eye laser surgery	2				
Ocular	Eye luxation	6		0.002		
Ocular	Eye movement disorder	433	1	0.176		
Ocular	Eye naevus	4		0.002		
Ocular	Eye oedema	200		0.081		
Ocular	Eye opacity	4		0.002		
Ocular	Eye operation	3				
Ocular	Eye pain	7690	13	3.119	0.017	182.36 [89.21–372.77]
Ocular	Eye patch application	1				
Ocular	Eye swelling	2138	14	0.867	0.018	47.08 [23.60–93.92]
Ocular	Eye symptom	33		0.013		
Ocular	Eye ulcer	15		0.006		
Ocular	Halo vision	34		0.014		
Ocular	Heteronymous diplopia	1				
Ocular	Homonymous diplopia	2				
Ocular	Intraocular haematoma	1				
Ocular	Intraocular lens implant	1				
Ocular	Intraocular pressure decreased	2				
Ocular	Intraocular pressure increased	81	3	0.033		
Ocular	Intraocular pressure test	18		0.007		
Ocular	Intraocular pressure test abnormal	3				
Ocular	Ocular discomfort	1112	4	0.451	0.005	85.70 [23.59–311.40]
Ocular	Ocular hyperaemia	1649	17	0.669	0.022	29.90 [15.96–56.03]
Ocular	Ocular hypertension	34		0.014		
Ocular	Ocular icterus	21		0.009		
Ocular	Ocular ischaemic syndrome	7		0.003		
Ocular	Ocular myasthenia	18		0.007		

Continues on the next page ... . . .

**CTC**	**Reaction**	**COVID**	**Influenza**	R_c	R_n	RR 99% CI
Ocular	Ocular vascular disorder	44		0.018		
Ocular	Optic disc haemorrhage	7		0.003		
Ocular	Periorbital haemorrhage	19		0.008		
Ocular	Photophobia	3216	9	1.304	0.012	110.16 [46.62–260.27]
Ocular	Retinal aneurysm	6		0.002		
Ocular	Retinal artery embolism	17		0.007		
Ocular	Retinal artery occlusion	242	1	0.098		
Ocular	Retinal cyst	2				
Ocular	Retinal degeneration	5		0.002		
Ocular	Retinal deposits	1				
Ocular	Retinal detachment	211		0.086		
Ocular	Retinal disorder	16		0.006		
Ocular	Retinal drusen	3				
Ocular	Retinal exudates	35		0.014		
Ocular	Retinal fovea disorder	3				
Ocular	Retinal function test abnormal	3				
Ocular	Retinal haemorrhage	140		0.057		
Ocular	Retinal infarction	5		0.002		
Ocular	Retinal injury	21		0.009		
Ocular	Retinal laser coagulation	1				
Ocular	Retinal migraine	115		0.047		
Ocular	Retinal neovascularisation	2				
Ocular	Retinal oedema	18		0.007		
Ocular	Retinal pallor	1				
Ocular	Retinal pigment epitheliopathy	6		0.002		
Ocular	Retinal tear	76		0.031		
Ocular	Retinal toxicity	8		0.003		
Ocular	Retinal vascular disorder	13		0.005		
Ocular	Retinal vascular occlusion	26		0.011		
Ocular	Retinal vasculitis	10		0.004		
Ocular	Retinal vein occlusion	546	1	0.221		
Ocular	Retinal vein varices	2				
Ocular	Retinal vessel avulsion	1				
Ocular	Tunnel vision	224		0.091		
Ocular	Vision blurred	8871	23	3.598	0.030	118.90 [69.44–203.58]
Localised pain	Administration site joint pain	16		0.006		
Localised pain	Administration site pain	2323	25	0.942	0.033	28.64 [17.07–48.08]

Continues on the next page ... . . .

CTC	Reaction	COVID	Influenza	R_c	R_n	RR 99% CI
Localised pain	Application site joint pain	15		0.006		
Localised pain	Application site pain	11240	7	4.559	0.009	495.00 [186.92–1310.85]
Localised pain	Incision site pain	14		0.006		
Localised pain	Infusion site joint pain	4	1	0.002		
Localised pain	Infusion site pain	34		0.014		
Localised pain	Injection site joint pain	168		0.068		
Localised pain	Injection site pain	90119	558	36.554	0.734	49.79 [44.63–55.54]
Localised pain	Instillation site pain	5		0.002		
Localised pain	Puncture site pain	612	1	0.248		
Localised pain	Vaccination site joint pain	458	9	0.186	0.012	15.69 [6.59–37.33]
Localised pain	Vaccination site pain	50853	389	20.627	0.512	40.30 [35.35–45.94]
Localised pain	Vessel puncture site pain	1				
Non-localised pain	Abdominal pain	17128	66	6.948	0.087	80.00 [58.23–109.92]
Non-localised pain	Abdominal pain lower	1427	4	0.579	0.005	109.98 [30.28–399.41]
Non-localised pain	Abdominal pain upper	12720	42	5.160	0.055	93.36 [62.70–139.02]
Non-localised pain	Amplified musculoskeletal pain syndrome	5		0.002		
Non-localised pain	Arthralgia	127580	397	51.749	0.522	99.07 [87.04–112.76]
Non-localised pain	Axillary pain	5899	35	2.393	0.046	51.96 [33.57–80.41]
Non-localised pain	Back pain	18612	69	7.549	0.091	83.15 [60.95–113.45]
Non-localised pain	Bladder pain	150	3	0.061		
Non-localised pain	Bone pain	5545	22	2.249	0.029	77.70 [44.82–134.71]
Non-localised pain	Breakthrough pain	6		0.002		
Non-localised pain	Central pain syndrome	31	1	0.013		
Non-localised pain	Chest pain	23818	50	9.661	0.066	146.85 [101.98–211.47]
Non-localised pain	Complex regional pain syndrome	66		0.027		
Non-localised pain	Ear pain	5402	14	2.191	0.018	118.95 [59.70–236.99]
Non-localised pain	Eosinophilia myalgia syndrome	1				
Non-localised pain	External ear pain	36		0.015		
Non-localised pain	Eyelid pain	111	1	0.045		
Non-localised pain	Facial pain	1254	6	0.509	0.008	64.43 [22.45–184.87]
Non-localised pain	Fibromyalgia	661	2	0.268		
Non-localised pain	Flank pain	577	2	0.234		
Non-localised pain	Fracture pain	6		0.002		
Non-localised pain	Gastrointestinal pain	1172	4	0.475	0.005	90.32 [24.86–328.16]
Non-localised pain	Genito-pelvic pain/penetration disorder	2				
Non-localised pain	Gingival pain	457	1	0.185		
Non-localised pain	Groin pain	719	4	0.292	0.005	55.41 [15.23–201.60]

Continues on the next page ... . . .

CTC	Reaction	COVID	Influenza	R_c	R_n	RR 99% CI
Non-localised pain	Growing pains	29		0.012		
Non-localised pain	Hepatic pain	220	1	0.089		
Non-localised pain	Hernia pain	12		0.005		
Non-localised pain	Ischaemic limb pain	18		0.007		
Non-localised pain	Laryngeal pain	33	1	0.013		
Non-localised pain	Ligament pain	21		0.009		
Non-localised pain	Lip pain	189	1	0.077		
Non-localised pain	Lymph node pain	3594	15	1.458	0.020	73.86 [37.93–143.83]
Non-localised pain	Masticatory pain	8		0.003		
Non-localised pain	Musculoskeletal chest pain	1444	4	0.586	0.005	111.29 [30.64–404.16]
Non-localised pain	Musculoskeletal pain	5022	16	2.037	0.021	96.76 [50.77–184.42]
Non-localised pain	Myalgia	195046	550	79.115	0.724	109.32 [97.94–122.03]
Non-localised pain	Myalgia intercostal	28	2	0.011		
Non-localised pain	Myofascial pain syndrome	44		0.018		
Non-localised pain	Neck pain	10779	54	4.372	0.071	61.53 [43.30–87.44]
Non-localised pain	Neuromuscular pain	22		0.009		
Non-localised pain	Non-cardiac chest pain	134	1	0.054		
Non-localised pain	Oesophageal pain	76	1	0.031		
Non-localised pain	Oral pain	731	3	0.297		
Non-localised pain	Oropharyngeal pain	13317	112	5.402	0.147	36.65 [28.71–46.80]
Non-localised pain	Pain	48310	261	19.596	0.343	57.06 [48.63–66.95]
Non-localised pain	Pain in extremity	73198	418	29.691	0.550	53.98 [47.58–61.25]
Non-localised pain	Pain in jaw	1949	5	0.791	0.007	120.16 [37.92–380.81]
Non-localised pain	Pain of skin	2425	12	0.984	0.016	62.30 [29.56–131.28]
Non-localised pain	Painful respiration	477	1	0.193		
Non-localised pain	Paroxysmal extreme pain disorder	19		0.008		
Non-localised pain	Patellofemoral pain syndrome	10		0.004		
Non-localised pain	Pelvic pain	637	1	0.258		
Non-localised pain	Perineal pain	13		0.005		
Non-localised pain	Periorbital pain	42		0.017		
Non-localised pain	Pleuritic pain	229		0.093		
Non-localised pain	Polymyalgia rheumatica	686	21	0.278	0.028	10.07 [5.69–17.82]
Non-localised pain	Post-traumatic pain	27		0.011		
Non-localised pain	Pulmonary pain	1014	4	0.411	0.005	78.15 [21.50–284.02]
Non-localised pain	Radicular pain	15	1	0.006		
Non-localised pain	Renal pain	2209	4	0.896	0.005	170.24 [46.91–617.89]
Non-localised pain	Salivary gland pain	55		0.022		

Continues on the next page ... . . .

CTC	Reaction	COVID	Influenza	R_c	R_n	RR 99% CI
Non-localised pain	Scar pain	62	1	0.025		
Non-localised pain	Sinus pain	910	5	0.369	0.007	56.11 [17.67–178.10]
Non-localised pain	Spinal pain	1074	5	0.436	0.007	66.22 [20.87–210.10]
Non-localised pain	Suprapubic pain	13	1	0.005		
Non-localised pain	Tendon pain	257	1	0.104		
Non-localised pain	Thyroid pain	56		0.023		
Non-localised pain	Tracheal pain	24		0.010		
Non-localised pain	Urinary tract pain	30		0.012		
Non-localised pain	Vascular pain	462		0.187		
Non-localised pain	Visceral pain	16		0.006		
Pulmonary	Acute pulmonary oedema	117		0.047		
Pulmonary	Acute respiratory distress syndrome	181	3	0.073		
Pulmonary	Acute respiratory failure	509	4	0.206	0.005	39.23 [10.77–142.93]
Pulmonary	Angiogram pulmonary abnormal	122		0.049		
Pulmonary	Apnoea	166		0.067		
Pulmonary	Cardio-respiratory distress	6		0.002		
Pulmonary	Cardiopulmonary failure	46		0.019		
Pulmonary	Chronic respiratory failure	13		0.005		
Pulmonary	Combined pulmonary fibrosis and emphysema	1				
Pulmonary	Cough	18855	160	7.648	0.211	36.33 [29.61–44.57]
Pulmonary	Dyspnoea	43967	175	17.834	0.230	77.45 [63.72–94.14]
Pulmonary	Dyspnoea at rest	188		0.076		
Pulmonary	Dyspnoea exertional	1660	3	0.673		
Pulmonary	Dyspnoea paroxysmal nocturnal	10		0.004		
Pulmonary	Eosinophilic pleural effusion	1				
Pulmonary	Hyperventilation	902	4	0.366	0.005	69.52 [19.12–252.73]
Pulmonary	Hypoxia	985	8	0.400	0.011	37.96 [15.21–94.71]
Pulmonary	Idiopathic pulmonary fibrosis	18		0.007		
Pulmonary	Infantile apnoea	1				
Pulmonary	Infectious pleural effusion	12		0.005		
Pulmonary	Laryngeal dyspnoea	8		0.003		
Pulmonary	Lower respiratory tract congestion	18		0.007		
Pulmonary	Malignant pleural effusion	2				
Pulmonary	Neonatal dyspnoea	2				
Pulmonary	Neonatal respiratory distress	5		0.002		
Pulmonary	Neonatal respiratory distress syndrome	3				

Continues on the next page ... . . .

CTC	Reaction	COVID	Influenza	R_c	R_n	RR 99% CI
Pulmonary	Neonatal respiratory failure	1				
Pulmonary	Nocturnal dyspnoea	30		0.012		
Pulmonary	Non-cardiogenic pulmonary oedema	1				
Pulmonary	Pleural effusion	774	3	0.314		
Pulmonary	Pneumonia	3411	31	1.384	0.041	33.92 [21.31–53.99]
Pulmonary	Pneumonitis	293	3	0.119		
Pulmonary	Pneumonitis chemical	1				
Pulmonary	Pneumothorax	147	1	0.060		
Pulmonary	Pneumothorax spontaneous	19		0.008		
Pulmonary	Productive cough	1188	14	0.482	0.018	26.16 [13.09–52.28]
Pulmonary	Pulmonary congestion	282	1	0.114		
Pulmonary	Pulmonary fibrosis	103	1	0.042		
Pulmonary	Pulmonary hypertension	94		0.038		
Pulmonary	Pulmonary infarction	265	2	0.107		
Pulmonary	Pulmonary oedema	552	3	0.224		
Pulmonary	Respiratory arrest	463	1	0.188		
Pulmonary	Respiratory disorder	845	2	0.343		
Pulmonary	Respiratory disorder neonatal	1				
Pulmonary	Respiratory distress	2433	13	0.987	0.017	57.69 [28.19–118.09]
Pulmonary	Respiratory failure	840	17	0.341	0.022	15.23 [8.10–28.63]
Pulmonary	Respiratory fatigue	97		0.039		
Pulmonary	Respiratory fume inhalation disorder	2				
Pulmonary	Respiratory gas exchange disorder	2				
Pulmonary	Respiratory rate	15		0.006		
Pulmonary	Respiratory rate decreased	81		0.033		
Pulmonary	Respiratory rate increased	432	1	0.175		
Pulmonary	Respiratory symptom	290		0.118		
Pulmonary	Respiratory tract congestion	232	1	0.094		
Pulmonary	Severe acute respiratory syndrome	38	2	0.015		
Pulmonary	Sleep apnoea syndrome	104	2	0.042		
Pulmonary	Upper respiratory tract congestion	54		0.022		
Renal/Genitourinary	Acute kidney injury	965	10	0.391	0.013	29.75 [13.12–67.46]
Renal/Genitourinary	Blood urine	83	1	0.034		
Renal/Genitourinary	Blood urine present	372	2	0.151		
Renal/Genitourinary	Cortisol free urine abnormal	1				
Renal/Genitourinary	Creatinine renal clearance abnormal	2				
Renal/Genitourinary	Dysuria	646	1	0.262		

Continues on the next page ... . . .

CTC	Reaction	COVID	Influenza	R_c	R_n	RR 99% CI
Renal/Genitourinary	Haemorrhagic adrenal infarction	5		0.002		
Renal/Genitourinary	Hepatorenal failure	2				
Renal/Genitourinary	pH urine abnormal	1				
Renal/Genitourinary	Prerenal failure	11		0.004		
Renal/Genitourinary	Renal failure	552	5	0.224	0.007	34.03 [10.70–108.26]
Renal/Genitourinary	Renal function test abnormal	20		0.008		
Renal/Genitourinary	Renal impairment	301	5	0.122	0.007	18.56 [5.81–59.29]
Renal/Genitourinary	Renal infarct	118	1	0.048		
Renal/Genitourinary	Urine abnormality	84		0.034		
Renal/Genitourinary	Urine analysis abnormal	51		0.021		
Renal/Genitourinary	Urine odour abnormal	118	1	0.048		
Sexual organs	Abnormal uterine bleeding	61		0.025		
Sexual organs	Abortion	56		0.023		
Sexual organs	Abortion complete	3				
Sexual organs	Abortion complicated	1				
Sexual organs	Abortion early	8		0.003		
Sexual organs	Abortion incomplete	2				
Sexual organs	Abortion induced	24	1	0.010		
Sexual organs	Abortion induced complete	1				
Sexual organs	Abortion infected	1				
Sexual organs	Abortion late	1				
Sexual organs	Abortion missed	86		0.035		
Sexual organs	Abortion of ectopic pregnancy	3				
Sexual organs	Abortion spontaneous	1937	3	0.786		
Sexual organs	Abortion spontaneous complete	6		0.002		
Sexual organs	Abortion spontaneous incomplete	3				
Sexual organs	Abortion threatened	12		0.005		
Sexual organs	Adnexa uteri pain	382		0.155		
Sexual organs	Amenorrhoea	4994	1	2.026		
Sexual organs	Dysfunctional uterine bleeding	1				
Sexual organs	Erection increased	17		0.007		
Sexual organs	Heavy menstrual bleeding	14319		5.808		
Sexual organs	Imminent abortion	1				
Sexual organs	Induced abortion failed	1				
Sexual organs	Infertility	28		0.011		
Sexual organs	Infertility female	8		0.003		
Sexual organs	Infertility male	3				

Continues on the next page ... . . .

CTC	Reaction	COVID	Influenza	R_c	R_n	RR 99% CI
Sexual organs	Intermenstrual bleeding	5714	1	2.318		
Sexual organs	Menstrual disorder	7935		3.219		
Sexual organs	Ovarian haemorrhage	13		0.005		
Sexual organs	Penile haemorrhage	21		0.009		
Sexual organs	Penile pain	14		0.006		
Sexual organs	Post abortion haemorrhage	1				
Sexual organs	Postmenopausal haemorrhage	1739		0.705		
Sexual organs	Premature menarche	11		0.004		
Sexual organs	Premenstrual pain	336		0.136		
Sexual organs	Premenstrual syndrome	394		0.160		
Sexual organs	Priapism	28		0.011		
Sexual organs	Spontaneous penile erection	13		0.005		
Sexual organs	Testicular pain	257		0.104		
Sexual organs	Testicular swelling	65		0.026		
Sexual organs	Uterine haemorrhage	208		0.084		
Sexual organs	Uterine pain	166		0.067		
Sexual organs	Vaginal discharge	285		0.116		
Sexual organs	Vaginal haemorrhage	3574	2	1.450		
Sexual organs	Vulvovaginal dryness	41		0.017		
Sexual organs	Vulvovaginal pain	109	1	0.044		
Thrombosis	Adrenal thrombosis	3				
Thrombosis	Aortic thrombosis	106		0.043		
Thrombosis	Arterial bypass thrombosis	1				
Thrombosis	Arterial thrombosis	148	1	0.060		
Thrombosis	Arteriovenous fistula thrombosis	8		0.003		
Thrombosis	Arteriovenous graft thrombosis	1				
Thrombosis	Atrial thrombosis	30		0.012		
Thrombosis	Axillary vein thrombosis	39		0.016		
Thrombosis	Basilar artery thrombosis	47		0.019		
Thrombosis	Brachiocephalic vein thrombosis	13		0.005		
Thrombosis	Brain stem thrombosis	16		0.006		
Thrombosis	Cardiac ventricular thrombosis	60		0.024		
Thrombosis	Carotid artery thrombosis	93		0.038		
Thrombosis	Catheter site thrombosis	2				
Thrombosis	Cavernous sinus thrombosis	34		0.014		
Thrombosis	Cerebellar artery thrombosis	8		0.003		
Thrombosis	Cerebral artery thrombosis	100		0.041		

Continues on the next page ... . . .

CTC	Reaction	COVID	Influenza	R_c	R_n	RR 99% CI
Thrombosis	Cerebral thrombosis	570	1	0.231		
Thrombosis	Cerebral venous sinus thrombosis	1229		0.499		
Thrombosis	Cerebral venous thrombosis	423		0.172		
Thrombosis	Coronary artery thrombosis	153		0.062		
Thrombosis	Coronary bypass thrombosis	2				
Thrombosis	Deep vein thrombosis	8608	5	3.492	0.007	530.72 [167.66–1679.95]
Thrombosis	Deep vein thrombosis postoperative	1				
Thrombosis	Device related thrombosis	4		0.002		
Thrombosis	Foetal placental thrombosis	1				
Thrombosis	Graft thrombosis	2				
Thrombosis	Hepatic artery thrombosis	8		0.003		
Thrombosis	Hepatic vascular thrombosis	12		0.005		
Thrombosis	Hepatic vein thrombosis	66		0.027		
Thrombosis	Infective thrombosis	3				
Thrombosis	Infusion site thrombosis	1				
Thrombosis	Injection site thrombosis	10		0.004		
Thrombosis	Jugular vein thrombosis	171		0.069		
Thrombosis	Mesenteric artery thrombosis	46		0.019		
Thrombosis	Mesenteric vein thrombosis	236		0.096		
Thrombosis	Ophthalmic artery thrombosis	17		0.007		
Thrombosis	Ophthalmic vein thrombosis	109		0.044		
Thrombosis	Ovarian vein thrombosis	30		0.012		
Thrombosis	Pelvic venous thrombosis	136		0.055		
Thrombosis	Penile vein thrombosis	14		0.006		
Thrombosis	Peripheral artery thrombosis	227		0.092		
Thrombosis	Portal vein thrombosis	461		0.187		
Thrombosis	Portosplenomesenteric venous thrombosis	43		0.017		
Thrombosis	Post thrombotic syndrome	8		0.003		
Thrombosis	Postoperative thrombosis	4		0.002		
Thrombosis	Postpartum thrombosis	1				
Thrombosis	Postpartum venous thrombosis	2				
Thrombosis	Precerebral artery thrombosis	1				
Thrombosis	Prosthetic cardiac valve thrombosis	11		0.004		
Thrombosis	Pulmonary artery thrombosis	58		0.024		
Thrombosis	Pulmonary thrombosis	624	3	0.253		
Thrombosis	Pulmonary venous thrombosis	17		0.007		
Thrombosis	Renal artery thrombosis	23		0.009		

Continues on the next page ... . . .

CTC	Reaction	COVID	Influenza	R_c	R_n	RR 99% CI
Thrombosis	Renal vascular thrombosis	13		0.005		
Thrombosis	Renal vein thrombosis	47		0.019		
Thrombosis	Renal-limited thrombotic microangiopathy	3				
Thrombosis	Retinal artery thrombosis	48		0.019		
Thrombosis	Retinal vascular thrombosis	88		0.036		
Thrombosis	Retinal vein thrombosis	172	1	0.070		
Thrombosis	Shunt thrombosis	1				
Thrombosis	Spinal artery thrombosis	2				
Thrombosis	Splenic artery thrombosis	21		0.009		
Thrombosis	Splenic thrombosis	26		0.011		
Thrombosis	Splenic vein thrombosis	87		0.035		
Thrombosis	Subclavian artery thrombosis	12		0.005		
Thrombosis	Subclavian vein thrombosis	107		0.043		
Thrombosis	Superior sagittal sinus thrombosis	141		0.057		
Thrombosis	Thrombosis	8067	6	3.272	0.008	414.47 [144.75–1186.75]
Thrombosis	Thrombosis in device	75		0.030		
Thrombosis	Thrombosis mesenteric vessel	27		0.011		
Thrombosis	Thrombosis prophylaxis	4		0.002		
Thrombosis	Thrombotic cerebral infarction	41		0.017		
Thrombosis	Thrombotic microangiopathy	32		0.013		
Thrombosis	Thrombotic stroke	53		0.021		
Thrombosis	Thrombotic thrombocytopenic purpura	135		0.055		
Thrombosis	Transverse sinus thrombosis	108		0.044		
Thrombosis	Truncus coeliacus thrombosis	8		0.003		
Thrombosis	Tumour thrombosis	2				
Thrombosis	Umbilical cord thrombosis	1				
Thrombosis	Vaccination site thrombosis	12		0.005		
Thrombosis	Vascular access site thrombosis	2				
Thrombosis	Vascular graft thrombosis	15		0.006		
Thrombosis	Vascular stent thrombosis	31		0.013		
Thrombosis	Vena cava thrombosis	56		0.023		
Thrombosis	Venous thrombosis	636		0.258		
Thrombosis	Venous thrombosis limb	718		0.291		
Thrombosis	Vertebral artery thrombosis	15		0.006		
Thrombosis	Visceral venous thrombosis	53		0.021		

Table 3: Vaccine-related risk estimates of influenza (R_n) and COVID-19 vaccines (R_c) per 100,000 exposed individuals by Common Toxicity Criteria (CTC) and adverse reactions in the VAERS database. Denominators of R_n and R_c: 3.61×10^8 and $205,482,061$ exposed individuals age 18 and older, respectively. Reactions without cases are left blank. Relative risks estimated only if at least 4 cases per reaction are available.

**CTC**	**Reaction**	**COVID cases**	**Influenza cases**	R_c	R_n	RR 99% CI
Allergic	Administration site pruritus	1				
Allergic	Allergic cough	4		0.002		
Allergic	Allergic respiratory disease	2				
Allergic	Allergic respiratory symptom	12	1	0.006		
Allergic	Anal pruritus	12	1	0.006		
Allergic	Dermatitis allergic	211	2	0.103		
Allergic	Dermatitis contact	149	4	0.073	0.001	65.44 [17.74–241.35]
Allergic	Ear pruritus	210	7	0.102	0.002	52.71 [19.59–141.79]
Allergic	Eye pruritus	1128	39	0.549	0.011	50.81 [33.40–77.30]
Allergic	Eyelids pruritus	103		0.050		
Allergic	Gingival pruritus	14	1	0.007		
Allergic	Hyperpyrexia	39		0.019		
Allergic	Injection site pruritus	17899	196	8.711	0.054	160.44 [133.34–193.04]
Allergic	Lip pruritus	326	6	0.159	0.002	95.46 [33.03–275.85]
Allergic	Nasal pruritus	76		0.037		
Allergic	Oral pruritus	448	7	0.218	0.002	112.44 [42.15–299.93]
Allergic	Pruritus	28088	514	13.669	0.142	96.00 [85.61–107.67]
Allergic	Pruritus allergic	3				
Allergic	Pruritus genital	36	1	0.018		
Allergic	Pyrexia	83571	1027	40.671	0.284	142.96 [131.86–155.00]
Allergic	Rash	29889	552	14.546	0.153	95.13 [85.16–106.26]
Allergic	Rash erythematous	8805	141	4.285	0.039	109.71 [88.16–136.52]
Allergic	Rash macular	3466	53	1.687	0.015	114.89 [80.44–164.10]
Allergic	Rash maculo-papular	253	8	0.123	0.002	55.56 [22.03–140.11]
Allergic	Rash maculovesicular	1				
Allergic	Rash morbilliform	142	1	0.069		
Allergic	Rash papular	2714	47	1.321	0.013	101.45 [69.45–148.19]
Allergic	Rash papulosquamous	1				
Allergic	Rash pruritic	7539	101	3.669	0.028	131.14 [101.31–169.74]
Allergic	Rash pustular	127	3	0.062		

Continues on the next page ... . . .

CTC	Reaction	COVID	Influenza	R_c	R_n	RR 99% CI
Allergic	Rash scarlatiniform	4		0.002		
Allergic	Rash vesicular	537	10	0.261	0.003	94.34 [41.46–214.65]
Allergic	Tongue pruritus	457	6	0.222	0.002	133.81 [46.43–385.64]
Allergic	Urticaria	16783	465	8.168	0.129	63.41 [56.18–71.57]
Allergic	Vaccination site pruritus	3283	1	1.598		
Allergic	Vulvovaginal pruritus	38	3	0.018		
Allergic	Implant site pruritus	1				
Allergic	Incision site pruritus	1				
Allergic	Neuropathic pruritus	1				
Arrythmia	Arrhythmia	662	1	0.322		
Arrythmia	Arrhythmia supraventricular	4		0.002		
Arrythmia	Arrhythmogenic right ventricular dysplasia	1				
Arrythmia	Atrial tachycardia	28	1	0.014		
Arrythmia	Bradycardia	537	2	0.261		
Arrythmia	Bradycardia foetal	3				
Arrythmia	Nodal arrhythmia	7		0.003		
Arrythmia	Palpitations	11182	85	5.442	0.024	231.12 [174.60–305.94]
Arrythmia	Postural orthostatic tachycardia syndrome	127	3	0.062		
Arrythmia	Presyncope	2478	26	1.206	0.007	167.44 [100.77–278.23]
Arrythmia	Respiratory sinus arrhythmia magnitude abnormal	3				
Arrythmia	Sinus arrhythmia	69	1	0.034		
Arrythmia	Sinus bradycardia	119		0.058		
Arrythmia	Sinus tachycardia	402	4	0.196	0.001	176.56 [48.39–644.18]
Arrythmia	Supraventricular tachycardia	317		0.154		
Arrythmia	Syncope	10956	221	5.332	0.061	87.09 [73.11–103.75]
Arrythmia	Tachyarrhythmia	16		0.008		
Arrythmia	Tachycardia	4696	40	2.285	0.011	206.25 [137.02–310.48]
Arrythmia	Ventricular arrhythmia	16		0.008		
Arrythmia	Ventricular tachyarrhythmia	2				
Arrythmia	Ventricular tachycardia	157		0.076		
Arrythmia	Pacemaker generated arrhythmia	2				
Arrythmia	Foetal arrhythmia	1				
Arrythmia	Neonatal tachycardia	1				
Arrythmia	Tachycardia foetal	4		0.002		
Arrythmia	Respiratory sinus arrhythmia magnitude	1				
Haematological	Granulocyte count	17		0.008		

Continues on the next page ... . . .

CTC	Reaction	COVID	Influenza	R_c	R_n	RR 99% CI
Haematological	Granulocyte count decreased	4		0.002		
Haematological	Granulocyte count increased	6	1	0.003		
Haematological	Granulocyte percentage	29		0.014		
Haematological	Lymphocyte count	138	3	0.067		
Haematological	Lymphocyte count abnormal	8		0.004		
Haematological	Lymphocyte count decreased	175	2	0.085		
Haematological	Lymphocyte count increased	85		0.041		
Haematological	Lymphocyte count normal	167	1	0.081		
Haematological	Lymphocyte percentage	140	1	0.068		
Haematological	Lymphocyte percentage abnormal	2				
Haematological	Lymphocyte percentage decreased	212		0.103		
Haematological	Lymphocyte percentage increased	49		0.024		
Haematological	Lymphocytic infiltration	6		0.003		
Haematological	Lymphocytosis	11		0.005		
Haematological	Lymphopenia	43		0.021		
Haematological	Neutrophil count	150		0.073		
Haematological	Neutrophil count abnormal	9		0.004		
Haematological	Neutrophil count decreased	139	1	0.068		
Haematological	Neutrophil count increased	209	5	0.102	0.001	73.44 [22.89–235.58]
Haematological	Neutrophil count normal	156	1	0.076		
Haematological	Neutrophil percentage	119	1	0.058		
Haematological	Neutrophil percentage decreased	66		0.032		
Haematological	Neutrophil percentage increased	210		0.102		
Haematological	Neutrophil toxic granulation present	2				
Haematological	Neutrophil/lymphocyte ratio	1				
Haematological	Neutrophilia	16		0.008		
Haematological	Granulocytopenia	1				
Haematological	Lymphocele	1				
Haematological	Lymphocyte morphology abnormal	12		0.006		
Haematological	Neutrophil percentage abnormal	2				
Haematological	Granulocytes abnormal	3				
Haematological	Lymphocytic leukaemia	1				
Haematological	Neutrophil function test abnormal	1				
Haematological	Neutrophilic dermatosis	2				
Cardiovascular	Accelerated hypertension	8		0.004		
Cardiovascular	Acute left ventricular failure	41		0.020		
Cardiovascular	Acute myocardial infarction	639	1	0.311		

Continues on the next page ... . . .

CTC	Reaction	COVID	Influenza	R_c	R_n	RR 99% CI
Cardiovascular	Autoimmune myocarditis	1				
Cardiovascular	Brain stem ischaemia	2				
Cardiovascular	Cardio-respiratory arrest	233		0.113		
Cardiovascular	Cerebral ischaemia	31		0.015		
Cardiovascular	Chronic left ventricular failure	34		0.017		
Cardiovascular	Diastolic hypertension	2				
Cardiovascular	ECG signs of myocardial ischaemia	1				
Cardiovascular	Hypertension	5127	43	2.495	0.012	209.47 [141.19–310.77]
Cardiovascular	Hypotension	3044	24	1.481	0.007	222.83 [131.44–377.76]
Cardiovascular	Infarction	29		0.014		
Cardiovascular	Intestinal ischaemia	32		0.016		
Cardiovascular	Ischaemia	46		0.022		
Cardiovascular	Ischaemic cardiomyopathy	13		0.006		
Cardiovascular	Ischaemic stroke	272	1	0.132		
Cardiovascular	Left ventricular dilatation	15		0.007		
Cardiovascular	Left ventricular dysfunction	91		0.044		
Cardiovascular	Left ventricular end-diastolic pressure decreased	1				
Cardiovascular	Left ventricular end-diastolic pressure increased	11		0.005		
Cardiovascular	Left ventricular enlargement	10		0.005		
Cardiovascular	Left ventricular failure	47		0.023		
Cardiovascular	Left ventricular hypertrophy	68		0.033		
Cardiovascular	Myocardial infarction	1066	4	0.519	0.001	468.20 [128.84–1701.43]
Cardiovascular	Myocardial ischaemia	44		0.021		
Cardiovascular	Myocarditis	1287	1	0.626		
Cardiovascular	Orthostatic hypertension	15		0.007		
Cardiovascular	Orthostatic hypotension	147	2	0.072		
Cardiovascular	Pericardial effusion	393	2	0.191		
Cardiovascular	Pericarditis	1069	3	0.520		
Cardiovascular	Peripheral ischaemia	26		0.013		
Cardiovascular	Phlebitis	44	2	0.021		
Cardiovascular	Phlebitis superficial	9		0.004		
Cardiovascular	Pulmonary embolism	2498	3	1.216		
Cardiovascular	Retinal ischaemia	3				
Cardiovascular	Silent myocardial infarction	2				
Cardiovascular	Sudden cardiac death	30		0.015		

Continues on the next page ... . . .

CTC	Reaction	COVID	Influenza	R_c	R_n	RR 99% CI
Cardiovascular	Systolic hypertension	3				
Cardiovascular	Transient ischaemic attack	519	6	0.253	0.002	151.97 [52.77–437.60]
Cardiovascular	Troponin	1323	9	0.644	0.002	258.26 [109.12–611.23]
Cardiovascular	Troponin abnormal	21		0.010		
Cardiovascular	Troponin C	1				
Cardiovascular	Troponin decreased	8		0.004		
Cardiovascular	Troponin I	293	3	0.143		
Cardiovascular	Troponin I abnormal	2				
Cardiovascular	Troponin I decreased	5		0.002		
Cardiovascular	Troponin I increased	206		0.100		
Cardiovascular	Troponin I normal	195		0.095		
Cardiovascular	Troponin increased	1283	5	0.624	0.001	450.81 [142.15–1429.71]
Cardiovascular	Troponin normal	727	5	0.354	0.001	255.45 [80.41–811.52]
Cardiovascular	Troponin T	74		0.036		
Cardiovascular	Troponin T increased	55	1	0.027		
Cardiovascular	Troponin T normal	38	1	0.018		
Cardiovascular	Viral myocarditis	12		0.006		
Cardiovascular	Viral pericarditis	8		0.004		
Cardiovascular	Eosinophilic myocarditis	3				
Cardiovascular	Left ventricular end-diastolic pressure	7		0.003		
Cardiovascular	Pericarditis constrictive	6		0.003		
Cardiovascular	Septic pulmonary embolism	1				
Cardiovascular	Spleen ischaemia	1				
Cardiovascular	ECG signs of myocardial infarction	3				
Cardiovascular	Giant cell myocarditis	1				
Cardiovascular	Hepatic ischaemia	2				
Cardiovascular	Purulent pericarditis	1				
Cardiovascular	Vaccination site ischaemia	1				
Cardiovascular	Left ventricular false tendon	1				
Coagulation	Activated partial thromboplastin time	291	7	0.142	0.002	73.03 [27.27–195.61]
Coagulation	Activated partial thromboplastin time prolonged	65		0.032		
Coagulation	Activated partial thromboplastin time ratio	1				
Coagulation	Activated partial thromboplastin time shortened	97		0.047		
Coagulation	Coagulation test abnormal	13		0.006		

Continues on the next page ... . . .

CTC	Reaction	COVID	Influenza	R_c	R_n	RR 99% CI
Coagulation	Coagulation time abnormal	2				
Coagulation	Coagulation time prolonged	7		0.003		
Coagulation	Coagulopathy	121		0.059		
Coagulation	Disseminated intravascular coagulation	39		0.019		
Coagulation	Fibrin D dimer	1139	4	0.554	0.001	500.26 [137.68–1817.66]
Coagulation	Fibrin D dimer decreased	9		0.004		
Coagulation	Fibrin D dimer increased	1024	2	0.498		
Coagulation	Fibrin degradation products	3				
Coagulation	Fibrin degradation products normal	1				
Coagulation	Hypercoagulation	37		0.018		
Coagulation	Immune thrombocytopenia	272	2	0.132		
Coagulation	Platelet count	404	3	0.197		
Coagulation	Platelet count abnormal	10	1	0.005		
Coagulation	Platelet count decreased	1232	6	0.600	0.002	360.74 [125.72–1035.14]
Coagulation	Platelet count increased	223	1	0.109		
Coagulation	Platelet disorder	33		0.016		
Coagulation	Prothrombin level	48		0.023		
Coagulation	Prothrombin time	352	5	0.171	0.001	123.68 [38.77–394.58]
Coagulation	Prothrombin time prolonged	132		0.064		
Coagulation	Prothrombin time shortened	21		0.010		
Coagulation	Coagulation factor increased	1				
Coagulation	Coagulation factor VIII level increased	1				
Coagulation	Congenital hypercoagulation	1				
Coagulation	Prothrombin level decreased	3				
Coagulation	Prothrombin level increased	6		0.003		
Coagulation	Prothrombin time ratio	2				
Coagulation	Coagulation factor V level decreased	1				
Coagulation	Prothrombin time ratio abnormal	1				
Coagulation	Coagulation factor VII level decreased	1				
Coagulation	Fibrin degradation products increased	1				
Coagulation	ISTH score for disseminated intravascular coagulation	1				
Constitutional	Ascites	66		0.032		
Constitutional	Body temperature abnormal	133	5	0.065	0.001	46.73 [14.45–151.08]
Constitutional	Body temperature increased	6120	101	2.978	0.028	106.45 [82.21–137.84]
Constitutional	Chills	74587	802	36.299	0.222	163.39 [149.11–179.03]
Constitutional	Fatigue	84182	650	40.968	0.180	227.53 [205.59–251.82]

Continues on the next page ... . . .

CTC	Reaction	COVID	Influenza	R_c	R_n	RR 99% CI
Constitutional	Lethargy	5529	67	2.691	0.019	144.98 [105.64–198.97]
Constitutional	Malaise	15548	293	7.567	0.081	93.23 [80.09–108.52]
Constitutional	Mental fatigue	53	2	0.026		
Constitutional	Muscle fatigue	467	5	0.227	0.001	164.09 [51.54–522.44]
Constitutional	Sweating fever	10		0.005		
Dermatological	Dermatitis	300	3	0.146		
Dermatological	Erythema	19217	804	9.352	0.223	41.99 [38.27–46.07]
Dermatological	Erythema annulare	5		0.002		
Dermatological	Erythema induratum	4		0.002		
Dermatological	Erythema infectiosum	1				
Dermatological	Erythema marginatum	2				
Dermatological	Erythema multiforme	148	3	0.072		
Dermatological	Erythema nodosum	56		0.027		
Dermatological	Erythema of eyelid	110	2	0.054		
Dermatological	Erythematotelangiectatic rosacea	1				
Dermatological	Flushing	7636	69	3.716	0.019	194.42 [142.39–265.48]
Dermatological	Injection site erythema	25224	857	12.276	0.237	51.71 [47.28–56.55]
Dermatological	Injection site inflammation	899	51	0.438	0.014	30.97 [21.37–44.87]
Dermatological	Injection site reaction	4059	131	1.975	0.036	54.44 [43.31–68.42]
Dermatological	Injection site swelling	20490	799	9.972	0.221	45.05 [41.06–49.44]
Dermatological	Injection site warmth	13000	401	6.327	0.111	56.96 [49.98–64.90]
Dermatological	Erythema dyschromicum perstans	1				
Gastrointestinal	Dehydration	2060	21	1.003	0.006	172.34 [97.96–303.20]
Gastrointestinal	Diarrhoea	18734	238	9.117	0.066	138.29 [116.90–163.59]
Gastrointestinal	Duodenal ulcer	3				
Gastrointestinal	Dysgeusia	3702	19	1.802	0.005	342.31 [189.29–619.03]
Gastrointestinal	Dyspepsia	1333	14	0.649	0.004	167.28 [83.73–334.18]
Gastrointestinal	Dysphagia	4068	50	1.980	0.014	142.94 [99.08–206.21]
Gastrointestinal	Gastric ulcer	19		0.009		
Gastrointestinal	Gastritis	133	1	0.065		
Gastrointestinal	Ileus	28		0.014		
Gastrointestinal	Nausea	57894	721	28.175	0.200	141.07 [128.09–155.36]
Gastrointestinal	Necrotising ulcerative gingivostomatitis	1				
Gastrointestinal	Oesophagitis	47		0.023		
Gastrointestinal	Pancreatitis	156		0.076		
Gastrointestinal	Stomatitis	620	10	0.302	0.003	108.92 [47.92–247.58]
Gastrointestinal	Vomiting	22091	373	10.751	0.103	104.05 [90.96–119.03]

Continues on the next page ... . . .

CTC	Reaction	COVID	Influenza	R_c	R_n	RR 99% CI
Gastrointestinal	Oesophagitis ulcerative	1				
Haemorraghe	Adrenal haemorrhage	5		0.002		
Haemorraghe	Anal haemorrhage	23		0.011		
Haemorraghe	Basal ganglia haematoma	1				
Haemorraghe	Basal ganglia haemorrhage	14		0.007		
Haemorraghe	Basal ganglia infarction	24		0.012		
Haemorraghe	Basal ganglia stroke	23		0.011		
Haemorraghe	Brain stem haemorrhage	12		0.006		
Haemorraghe	Brain stem infarction	24		0.012		
Haemorraghe	Brain stem stroke	23		0.011		
Haemorraghe	Bullous haemorrhagic dermatosis	4		0.002		
Haemorraghe	Diarrhoea haemorrhagic	130	1	0.063		
Haemorraghe	Diverticulum intestinal haemorrhagic	4		0.002		
Haemorraghe	Embolic stroke	50		0.024		
Haemorraghe	Gastric haemorrhage	12		0.006		
Haemorraghe	Gastrointestinal haemorrhage	205	1	0.100		
Haemorraghe	Haemorrhage	682	21	0.332	0.006	57.06 [32.24–100.96]
Haemorraghe	Haemorrhage subcutaneous	18	2	0.009		
Haemorraghe	Haemorrhage subepidermal	3				
Haemorraghe	Haemorrhage urinary tract	108	1	0.053		
Haemorraghe	Haemorrhoid operation	7		0.003		
Haemorraghe	Haemorrhoidal haemorrhage	8		0.004		
Haemorraghe	Haemorrhoids	89	2	0.043		
Haemorraghe	Haemorrhoids thrombosed	18		0.009		
Haemorraghe	Intestinal haemorrhage	10		0.005		
Haemorraghe	Large intestinal haemorrhage	3				
Haemorraghe	Lower gastrointestinal haemorrhage	12		0.006		
Haemorraghe	Mouth haemorrhage	104	1	0.051		
Haemorraghe	Mucosal haemorrhage	7		0.003		
Haemorraghe	Petechiae	610	6	0.297	0.002	178.61 [62.08–513.86]
Haemorraghe	Pharyngeal haemorrhage	8		0.004		
Haemorraghe	Pulmonary haemorrhage	22		0.011		
Haemorraghe	Rectal haemorrhage	204		0.099		
Haemorraghe	Renal haemorrhage	8		0.004		
Haemorraghe	Respiratory tract haemorrhage	4		0.002		
Haemorraghe	Shock haemorrhagic	11		0.005		
Haemorraghe	Skin haemorrhage	76	1	0.037		

Continues on the next page ... . . .

CTC	Reaction	COVID	Influenza	R_c	R_n	RR 99% CI
Haemorraghe	Skin ulcer haemorrhage	3				
Haemorraghe	Small intestinal ulcer haemorrhage	1				
Haemorraghe	Spontaneous haemorrhage	12		0.006		
Haemorraghe	Subarachnoid haemorrhage	113	1	0.055		
Haemorraghe	Subdural haematoma	91	1	0.044		
Haemorraghe	Subdural haemorrhage	18		0.009		
Haemorraghe	Subdural hygroma	2				
Haemorraghe	Tongue haemorrhage	15		0.007		
Haemorraghe	Ulcer haemorrhage	13		0.006		
Haemorraghe	Upper gastrointestinal haemorrhage	23		0.011		
Haemorraghe	Urethral haemorrhage	7		0.003		
Haemorraghe	Urinary bladder haemorrhage	15		0.007		
Haemorraghe	Vaccination site haemorrhage	184	3	0.090		
Haemorraghe	Wound haemorrhage	28	1	0.014		
Haemorraghe	Gastric ulcer haemorrhage	3				
Haemorraghe	Intra-abdominal haemorrhage	2				
Haemorraghe	Pelvic haemorrhage	4		0.002		
Haemorraghe	Peptic ulcer haemorrhage	2				
Haemorraghe	Pericardial haemorrhage	8		0.004		
Haemorraghe	Spinal subdural haematoma	1				
Haemorraghe	Subdural haematoma evacuation	6		0.003		
Haemorraghe	Venous haemorrhage	2				
Haemorraghe	Duodenal ulcer haemorrhage	2				
Haemorraghe	Muscle haemorrhage	1				
Haemorraghe	Large intestinal ulcer haemorrhage	2				
Haemorraghe	Subarachnoid haematoma	1				
Haemorraghe	Rectal ulcer haemorrhage	1				
Haemorraghe	Spinal subarachnoid haemorrhage	1				
Neurological	Action tremor	3				
Neurological	Acute motor axonal neuropathy	3				
Neurological	Acute motor-sensory axonal neuropathy	9	1	0.004		
Neurological	Acute polyneuropathy	2				
Neurological	Administration site dysaesthesia	1				
Neurological	Anaesthesia	5		0.002		
Neurological	Anal hypoaesthesia	2				
Neurological	Aphasia	1378	13	0.671	0.004	186.23 [90.85–381.74]
Neurological	Ataxia	139	1	0.068		

Continues on the next page ... . . .

CTC	Reaction	COVID	Influenza	R_c	R_n	RR 99% CI
Neurological	Autoimmune neuropathy	1				
Neurological	Autonomic neuropathy	2				
Neurological	Axonal and demyelinating polyneuropathy	4	1	0.002		
Neurological	Axonal neuropathy	3				
Neurological	Cerebral amyloid angiopathy	3				
Neurological	Cerebral arteriosclerosis	4		0.002		
Neurological	Cerebral artery embolism	19		0.009		
Neurological	Cerebral artery occlusion	61		0.030		
Neurological	Cerebral artery stenosis	17		0.008		
Neurological	Cerebral atrophy	54		0.026		
Neurological	Cerebral calcification	10		0.005		
Neurological	Cerebral cavernous malformation	6		0.003		
Neurological	Cerebral circulatory failure	1				
Neurological	Cerebral congestion	15		0.007		
Neurological	Cerebral cyst	3				
Neurological	Cerebral disorder	60	1	0.029		
Neurological	Cerebral haematoma	18		0.009		
Neurological	Cerebral haemorrhage	333	1	0.162		
Neurological	Cerebral haemorrhage foetal	2				
Neurological	Cerebral hypoperfusion	4		0.002		
Neurological	Cerebral infarction	224		0.109		
Neurological	Cerebral mass effect	39		0.019		
Neurological	Cerebral microhaemorrhage	5		0.002		
Neurological	Cerebral palsy	13		0.006		
Neurological	Cerebral revascularisation	1				
Neurological	Cerebral small vessel ischaemic disease	77		0.037		
Neurological	Cerebral vascular occlusion	1				
Neurological	Cerebral vasoconstriction	4		0.002		
Neurological	Cervicogenic vertigo	1				
Neurological	Chronic inflammatory demyelinating polyradiculoneuropathy	40	12	0.019	0.003	5.86 [2.51–13.67]
Neurological	Confusional arousal	2				
Neurological	Cranial nerve paralysis	4		0.002		
Neurological	Delusion	114	1	0.055		
Neurological	Demyelinating polyneuropathy	25	2	0.012		
Neurological	Dental paraesthesia	15		0.007		
Neurological	Diabetic neuropathy	10	1	0.005		

Continues on the next page ... . . .

CTC	Reaction	COVID	Influenza	R_c	R_n	RR 99% CI
Neurological	Diaphragmatic paralysis	5	1	0.002		
Neurological	Diplegia	68	5	0.033	0.001	23.89 [7.24–78.82]
Neurological	Dizziness	58507	668	28.473	0.185	153.87 [139.20–170.10]
Neurological	Dizziness exertional	31		0.015		
Neurological	Dizziness postural	587	7	0.286	0.002	147.32 [55.33–392.29]
Neurological	Dysaesthesia	43	2	0.021		
Neurological	Dystonic tremor	1				
Neurological	Essential tremor	20		0.010		
Neurological	Extraocular muscle paresis	46		0.022		
Neurological	Facial paralysis	2092	44	1.018	0.012	83.53 [56.42–123.67]
Neurological	Facial paresis	390	7	0.190	0.002	97.88 [36.65–261.39]
Neurological	Familial periodic paralysis	1				
Neurological	Genital paraesthesia	7		0.003		
Neurological	Guillain-Barre syndrome	585	90	0.285	0.025	11.42 [8.53–15.29]
Neurological	Hemiparaesthesia	22		0.011		
Neurological	Hemiparesis	688	7	0.335	0.002	172.67 [64.90–459.39]
Neurological	Hemiplegia	225	2	0.109		
Neurological	Hypoaesthesia	17990	346	8.755	0.096	91.35 [79.43–105.05]
Neurological	Hypoaesthesia eye	121	1	0.059		
Neurological	Hypoaesthesia oral	4117	40	2.004	0.011	180.82 [120.09–272.26]
Neurological	Hypoaesthesia teeth	9		0.004		
Neurological	Hypoglossal nerve paresis	1				
Neurological	IIIrd nerve paralysis	29	1	0.014		
Neurological	Immune-mediated neuropathy	3				
Neurological	Initial insomnia	122	1	0.059		
Neurological	Injection site hypoaesthesia	603	22	0.293	0.006	48.15 [27.53–84.22]
Neurological	Injection site paraesthesia	543	17	0.264	0.005	56.12 [29.75–105.83]
Neurological	Insomnia	6044	112	2.941	0.031	94.81 [74.16–121.20]
Neurological	Instillation site paraesthesia	4		0.002		
Neurological	Intention tremor	6		0.003		
Neurological	Intranasal hypoaesthesia	15		0.007		
Neurological	IVth nerve paralysis	19		0.009		
Neurological	Memory impairment	1294	14	0.630	0.004	162.38 [81.27–324.44]
Neurological	Middle insomnia	64	6	0.031	0.002	18.74 [6.24–56.28]
Neurological	Mononeuropathy multiplex	1				
Neurological	Monoparesis	19		0.009		
Neurological	Monoplegia	229	10	0.111	0.003	40.23 [17.51–92.46]

Continues on the next page ... . . .

CTC	Reaction	COVID	Influenza	R_c	R_n	RR 99% CI
Neurological	Neuropathy peripheral	869	21	0.423	0.006	72.70 [41.16–128.40]
Neurological	Nystagmus	134	2	0.065		
Neurological	Ophthalmoplegia	28		0.014		
Neurological	Optic ischaemic neuropathy	39		0.019		
Neurological	Opticokinetic nystagmus tests	1				
Neurological	Opticokinetic nystagmus tests abnormal	1				
Neurological	Oral dysaesthesia	3				
Neurological	Palmar-plantar erythrodysaesthesia syndrome	4		0.002		
Neurological	Paraesthesia	19360	358	9.422	0.099	95.01 [82.81–109.00]
Neurological	Paraesthesia ear	17		0.008		
Neurological	Paraesthesia mucosal	2				
Neurological	Paraesthesia oral	5892	54	2.867	0.015	191.69 [134.80–272.60]
Neurological	Paralysis	425	13	0.207	0.004	57.44 [27.81–118.62]
Neurological	Paraparesis	1				
Neurological	Paraplegia	16	2	0.008		
Neurological	Paresis	10		0.005		
Neurological	Peripheral motor neuropathy	2	1			
Neurological	Peripheral paralysis	5		0.002		
Neurological	Peripheral sensory neuropathy	28	3	0.014		
Neurological	Persistent postural-perceptual dizziness	3				
Neurological	Pharyngeal hypoaesthesia	428	1	0.208		
Neurological	Pharyngeal paraesthesia	856	6	0.417	0.002	250.64 [87.25–720.03]
Neurological	Polyneuropathy	24		0.012		
Neurological	Polyneuropathy chronic	1				
Neurological	Postictal paralysis	1	1			
Neurological	Quadriplegia	12		0.006		
Neurological	Resting tremor	7		0.003		
Neurological	Sciatic nerve neuropathy	3				
Neurological	Seizure	3385	42	1.647	0.012	141.59 [94.92–211.21]
Neurological	Seizure cluster	7		0.003		
Neurological	Seizure like phenomena	369	13	0.180	0.004	49.87 [24.11–103.16]
Neurological	Sleep disorder due to general medical condition, insomnia type	4		0.002		
Neurological	Sleep paralysis	30		0.015		
Neurological	Small fibre neuropathy	65	2	0.032		

Continues on the next page … . . .

CTC	Reaction	COVID	Influenza	R_c	R_n	RR 99% CI
Neurological	Subacute inflammatory demyelinating polyneuropathy	13	1	0.006		
Neurological	Terminal insomnia	13		0.006		
Neurological	Tongue paralysis	19		0.009		
Neurological	Transient aphasia	6		0.003		
Neurological	Tremor	10951	214	5.329	0.059	89.90 [75.26–107.40]
Neurological	Vaccination site dysaesthesia	21		0.010		
Neurological	Vaccination site hypoaesthesia	52		0.025		
Neurological	Vaccination site paraesthesia	51		0.025		
Neurological	Vertigo	6237	65	3.035	0.018	168.58 [122.27–232.42]
Neurological	Vertigo positional	215	3	0.105		
Neurological	Vestibular nystagmus	2				
Neurological	VIth nerve paralysis	39		0.019		
Neurological	Vocal cord paralysis	28	1	0.014		
Neurological	Vocal cord paresis	7		0.003		
Neurological	Cardiac autonomic neuropathy	1				
Neurological	Cerebral artery perforation	1				
Neurological	Cerebral endovascular aneurysm repair	5		0.002		
Neurological	Cerebral haemangioma	1				
Neurological	Cerebral microangiopathy	7		0.003		
Neurological	Cerebral microembolism	2				
Neurological	Cerebral microinfarction	2				
Neurological	Cerebral ventricle collapse	1				
Neurological	Cerebral ventricle dilatation	7		0.003		
Neurological	Diabetic gastroparesis	1				
Neurological	Eye paraesthesia	4		0.002		
Neurological	Genital hypoaesthesia	6		0.003		
Neurological	Intranasal paraesthesia	3				
Neurological	Laryngeal tremor	1				
Neurological	Mononeuropathy	6	2	0.003		
Neurological	Multifocal motor neuropathy	3				
Neurological	Obturator neuropathy	1				
Neurological	Oculofacial paralysis	2				
Neurological	Optic neuropathy	8		0.004		
Neurological	Peripheral sensorimotor neuropathy	6	1	0.003		
Neurological	Postural tremor	2				
Neurological	Quadriparesis	2	3			

Continues on the next page ... . . .

CTC	Reaction	COVID	Influenza	R_c	R_n	RR 99% CI
Neurological	Vertigo CNS origin	4		0.002		
Neurological	Cerebral artery stent insertion	2				
Neurological	Cerebral reperfusion injury	1				
Neurological	Enteric neuropathy	1				
Neurological	Parkinsonian rest tremor	1				
Neurological	Seizure anoxic	1				
Neurological	Seizure prophylaxis	2				
Neurological	Sensory neuropathy hereditary	1				
Neurological	Hypoglossal nerve paralysis	1				
Neurological	IIIrd nerve paresis	1				
Neurological	Thermohypoaesthesia	1				
Neurological	Polyneuropathy idiopathic progressive	1				
Neurological	Trigeminal nerve paresis	1				
Ocular	Central vision loss	9	1	0.004		
Ocular	Colour vision tests	2				
Ocular	Diplopia	797	11	0.388	0.003	127.29 [58.24–278.23]
Ocular	Dry eye	438	8	0.213	0.002	96.19 [38.37–241.11]
Ocular	Eye abscess	1				
Ocular	Eye allergy	7		0.003		
Ocular	Eye colour change	24		0.012		
Ocular	Eye contusion	103	1	0.050		
Ocular	Eye discharge	171	25	0.083	0.007	12.02 [6.92–20.86]
Ocular	Eye disorder	378	2	0.184		
Ocular	Eye excision	1				
Ocular	Eye haematoma	4		0.002		
Ocular	Eye haemorrhage	263	1	0.128		
Ocular	Eye infarction	8		0.004		
Ocular	Eye infection	76	1	0.037		
Ocular	Eye infection bacterial	7		0.003		
Ocular	Eye infection viral	1				
Ocular	Eye inflammation	126	1	0.061		
Ocular	Eye injury	32		0.016		
Ocular	Eye irrigation	6	1	0.003		
Ocular	Eye irritation	918	32	0.447	0.009	50.40 [31.71–80.09]
Ocular	Eye laser surgery	10		0.005		
Ocular	Eye movement disorder	705	11	0.343	0.003	112.60 [51.48–246.29]
Ocular	Eye oedema	24	1	0.012		

Continues on the next page ... . . .

CTC	Reaction	COVID	Influenza	R_c	R_n	RR 99% CI
Ocular	Eye operation	12		0.006		
Ocular	Eye pain	2454	29	1.194	0.008	148.67 [91.89–240.53]
Ocular	Eye swelling	2407	68	1.171	0.019	62.19 [45.30–85.36]
Ocular	Eye symptom	4		0.002		
Ocular	Halo vision	13	1	0.006		
Ocular	Intraocular pressure increased	32	1	0.016		
Ocular	Intraocular pressure test	33	1	0.016		
Ocular	Intraocular pressure test abnormal	5		0.002		
Ocular	Ocular discomfort	699	4	0.340	0.001	307.01 [84.38–1117.08]
Ocular	Ocular hyperaemia	1125	45	0.547	0.012	43.92 [29.69–64.97]
Ocular	Ocular hypertension	6		0.003		
Ocular	Ocular icterus	15		0.007		
Ocular	Ocular vascular disorder	8		0.004		
Ocular	Photophobia	1546	10	0.752	0.003	271.61 [119.96–614.95]
Ocular	Retinal artery occlusion	74		0.036		
Ocular	Retinal degeneration	1				
Ocular	Retinal deposits	1				
Ocular	Retinal detachment	52	2	0.025		
Ocular	Retinal disorder	13		0.006		
Ocular	Retinal exudates	11	1	0.005		
Ocular	Retinal haemorrhage	39		0.019		
Ocular	Retinal migraine	35		0.017		
Ocular	Retinal oedema	7		0.003		
Ocular	Retinal operation	8		0.004		
Ocular	Retinal pigment epitheliopathy	2				
Ocular	Retinal scar	3				
Ocular	Retinal tear	31		0.015		
Ocular	Retinal vascular occlusion	8		0.004		
Ocular	Retinal vasculitis	7	1	0.003		
Ocular	Retinal vein occlusion	83		0.040		
Ocular	Tunnel vision	384	4	0.187	0.001	168.66 [46.21–615.52]
Ocular	Vision blurred	5502	65	2.678	0.018	148.71 [107.84–205.07]
Ocular	Colour vision tests abnormal	1				
Ocular	Colour vision tests abnormal red-green	1				
Ocular	Eye infection fungal	1				
Ocular	Eye infection staphylococcal	1				
Ocular	Eye muscle recession	1				

Continues on the next page ... . . .

CTC	Reaction	COVID	Influenza	R_c	R_n	RR 99% CI
Ocular	Eye ulcer	5		0.002		
Ocular	Intraocular pressure test normal	2				
Ocular	Periorbital haemorrhage	9		0.004		
Ocular	Retinal function test abnormal	4		0.002		
Ocular	Retinal function test normal	2				
Ocular	Retinal infarction	3				
Ocular	Retinal vascular disorder	4		0.002		
Ocular	Eye opacity	1				
Ocular	Eye patch application	5		0.002		
Ocular	Eye pH test	1				
Ocular	Ocular myasthenia	5		0.002		
Ocular	Retinal artery embolism	1				
Ocular	Intraocular pressure decreased	3				
Ocular	Retinal injury	1				
Ocular	Retinal pallor	1				
Localised pain	Administration site pain	15	5	0.007	0.001	5.27 [1.39–19.93]
Localised pain	Application site pain	6		0.003		
Localised pain	Incision site pain	7		0.003		
Localised pain	Injection site joint pain	121	9	0.059	0.002	23.62 [9.70–57.52]
Localised pain	Injection site pain	34340	1251	16.712	0.347	48.23 [44.78–51.94]
Localised pain	Instillation site pain	1				
Localised pain	Vaccination site joint pain	20	1	0.010		
Localised pain	Vaccination site pain	10614	32	5.165	0.009	582.72 [369.33–919.42]
Localised pain	Infusion site pain	3				
Localised pain	Puncture site pain	1				
Non-localised pain	Abdominal pain	5408	43	2.632	0.012	220.95 [148.95–327.77]
Non-localised pain	Abdominal pain lower	580	2	0.282		
Non-localised pain	Abdominal pain upper	5854	64	2.849	0.018	160.70 [116.25–222.13]
Non-localised pain	Arthralgia	33836	652	16.467	0.181	91.17 [82.34–100.95]
Non-localised pain	Axillary pain	4292	55	2.089	0.015	137.10 [96.66–194.46]
Non-localised pain	Back pain	10623	192	5.170	0.053	97.20 [80.58–117.26]
Non-localised pain	Bladder pain	71		0.035		
Non-localised pain	Bone pain	1835	20	0.893	0.006	161.19 [90.33–287.63]
Non-localised pain	Chest pain	15403	115	7.496	0.032	235.31 [184.90–299.46]
Non-localised pain	Complex regional pain syndrome	31	2	0.015		
Non-localised pain	Ear pain	2994	28	1.457	0.008	187.86 [115.19–306.35]
Non-localised pain	External ear pain	37		0.018		

Continues on the next page ... . . .

CTC	Reaction	COVID	Influenza	R_c	R_n	RR 99% CI
Non-localised pain	Eyelid pain	79	2	0.038		
Non-localised pain	Facial pain	1131	16	0.550	0.004	124.19 [64.93–237.53]
Non-localised pain	Fibromyalgia	262	5	0.128	0.001	92.06 [28.78–294.51]
Non-localised pain	Flank pain	503	13	0.245	0.004	67.98 [32.97–140.15]
Non-localised pain	Gastrointestinal pain	317	4	0.154	0.001	139.23 [38.10–508.84]
Non-localised pain	Genito-pelvic pain/penetration disorder	3				
Non-localised pain	Gingival pain	271	2	0.132		
Non-localised pain	Groin pain	389	5	0.189	0.001	136.68 [42.88–435.71]
Non-localised pain	Hepatic pain	66		0.032		
Non-localised pain	Ligament pain	21		0.010		
Non-localised pain	Lip pain	212	5	0.103	0.001	74.49 [23.22–238.92]
Non-localised pain	Lymph node pain	3710	44	1.806	0.012	148.13 [100.23–218.93]
Non-localised pain	Musculoskeletal chest pain	960	14	0.467	0.004	120.47 [60.22–241.00]
Non-localised pain	Musculoskeletal pain	405	82	0.197	0.023	8.68 [6.35–11.85]
Non-localised pain	Myalgia	34541	459	16.810	0.127	132.21 [117.14–149.22]
Non-localised pain	Myalgia intercostal	2				
Non-localised pain	Myofascial pain syndrome	6		0.003		
Non-localised pain	Neck pain	8710	200	4.239	0.055	76.51 [63.64–91.99]
Non-localised pain	Neuromuscular pain	8		0.004		
Non-localised pain	Non-cardiac chest pain	40	1	0.019		
Non-localised pain	Oesophageal pain	37	1	0.018		
Non-localised pain	Oral pain	540	8	0.263	0.002	118.59 [47.38–296.80]
Non-localised pain	Oropharyngeal pain	8686	116	4.227	0.032	131.55 [103.40–167.36]
Non-localised pain	Pain	72713	1473	35.387	0.408	86.72 [81.04–92.81]
Non-localised pain	Pain in extremity	52876	1551	25.733	0.430	59.89 [56.05–64.00]
Non-localised pain	Pain in jaw	1707	24	0.831	0.007	124.96 [73.59–212.18]
Non-localised pain	Pain of skin	1449	27	0.705	0.007	94.28 [57.17–155.50]
Non-localised pain	Painful respiration	542	7	0.264	0.002	136.03 [51.06–362.39]
Non-localised pain	Patellofemoral pain syndrome	2	1			
Non-localised pain	Pelvic pain	295	6	0.144	0.002	86.38 [29.86–249.87]
Non-localised pain	Perineal pain	6		0.003		
Non-localised pain	Periorbital pain	31	1	0.015		
Non-localised pain	Pleuritic pain	201	1	0.098		
Non-localised pain	Polymyalgia rheumatica	197	6	0.096	0.002	57.68 [19.84–167.74]
Non-localised pain	Pulmonary pain	408	3	0.199		
Non-localised pain	Radicular pain	11		0.005		
Non-localised pain	Renal pain	522	3	0.254		

Continues on the next page ... . . .

CTC	Reaction	COVID	Influenza	R_c	R_n	RR 99% CI
Non-localised pain	Salivary gland pain	16		0.008		
Non-localised pain	Scar pain	14		0.007		
Non-localised pain	Sinus pain	378	6	0.184	0.002	110.68 [38.35–319.43]
Non-localised pain	Spinal pain	570	20	0.277	0.006	50.07 [27.87–89.96]
Non-localised pain	Suprapubic pain	10		0.005		
Non-localised pain	Tendon pain	135	2	0.066		
Non-localised pain	Thyroid pain	22		0.011		
Non-localised pain	Urinary tract pain	14		0.007		
Non-localised pain	Vascular pain	81		0.039		
Non-localised pain	Visceral pain	14		0.007		
Non-localised pain	Central pain syndrome	5		0.002		
Non-localised pain	Eosinophilia myalgia syndrome	1				
Non-localised pain	Hernia pain	2				
Non-localised pain	Ischaemic limb pain	2				
Non-localised pain	Fracture pain	1				
Non-localised pain	Growing pains	3				
Non-localised pain	Laryngeal pain	4		0.002		
Non-localised pain	Tracheal pain	5		0.002		
Non-localised pain	Breakthrough pain	1				
Non-localised pain	Masticatory pain	1				
Pulmonary	Acute pulmonary oedema	19		0.009		
Pulmonary	Acute respiratory distress syndrome	144		0.070		
Pulmonary	Acute respiratory failure	931	3	0.453		
Pulmonary	Angiogram pulmonary abnormal	582		0.283		
Pulmonary	Apnoea	50	1	0.024		
Pulmonary	Apnoea test	5		0.002		
Pulmonary	Cardio-respiratory distress	4		0.002		
Pulmonary	Cardiopulmonary failure	10		0.005		
Pulmonary	Chronic respiratory failure	18		0.009		
Pulmonary	Cough	15388	168	7.489	0.047	160.92 [131.77–196.51]
Pulmonary	Dyspnoea	31027	336	15.100	0.093	162.23 [140.86–186.85]
Pulmonary	Dyspnoea at rest	20		0.010		
Pulmonary	Dyspnoea exertional	859	5	0.418	0.001	301.83 [95.06–958.28]
Pulmonary	Dyspnoea paroxysmal nocturnal	17		0.008		
Pulmonary	Hyperventilation	622	2	0.303		
Pulmonary	Hypoxia	1330	1	0.647		
Pulmonary	Idiopathic pulmonary fibrosis	4		0.002		

Continues on the next page ... . . .

CTC	Reaction	COVID	Influenza	R_c	R_n	RR 99% CI
Pulmonary	Infectious pleural effusion	5	1	0.002		
Pulmonary	Lower respiratory tract congestion	44	1	0.021		
Pulmonary	Pleural effusion	494		0.240		
Pulmonary	Pneumonia	1950	12	0.949	0.003	285.49 [135.41–601.88]
Pulmonary	Pneumonitis	145	2	0.071		
Pulmonary	Pneumothorax	92	1	0.045		
Pulmonary	Pneumothorax spontaneous	11	1	0.005		
Pulmonary	Productive cough	1101	17	0.536	0.005	113.78 [60.63–213.54]
Pulmonary	Pulmonary congestion	340	1	0.165		
Pulmonary	Pulmonary fibrosis	74	1	0.036		
Pulmonary	Pulmonary hypertension	83		0.040		
Pulmonary	Pulmonary infarction	84		0.041		
Pulmonary	Pulmonary oedema	355	3	0.173		
Pulmonary	Respiratory arrest	289	3	0.141		
Pulmonary	Respiratory disorder	198	4	0.096	0.001	86.96 [23.68–319.37]
Pulmonary	Respiratory distress	405	3	0.197		
Pulmonary	Respiratory failure	446	4	0.217	0.001	195.89 [53.72–714.24]
Pulmonary	Respiratory rate	166		0.081		
Pulmonary	Respiratory rate decreased	67	3	0.033		
Pulmonary	Respiratory rate increased	398	6	0.194	0.002	116.54 [40.40–336.19]
Pulmonary	Respiratory symptom	155	1	0.075		
Pulmonary	Respiratory tract congestion	2420	27	1.178	0.007	157.47 [95.65–259.22]
Pulmonary	Severe acute respiratory syndrome	7		0.003		
Pulmonary	Sleep apnoea syndrome	73	3	0.036		
Pulmonary	Upper respiratory tract congestion	93	1	0.045		
Pulmonary	Nocturnal dyspnoea	6		0.003		
Pulmonary	Respiratory fatigue	3				
Pulmonary	Eosinophilic pleural effusion	1				
Pulmonary	Non-cardiogenic pulmonary oedema	1				
Pulmonary	Neonatal respiratory distress syndrome	1				
Pulmonary	Pneumothorax traumatic	1				
Pulmonary	Respiratory gas exchange disorder	1				
Pulmonary	Malignant pleural effusion	2				
Pulmonary	Pneumonitis chemical	1				
Renal/Genitourinary	Acute kidney injury	989	2	0.481		
Renal/Genitourinary	Blood urine	50		0.024		
Renal/Genitourinary	Blood urine present	556	4	0.271	0.001	244.20 [67.05–889.39]

Continues on the next page ... . . .

CTC	Reaction	COVID	Influenza	R_c	R_n	RR 99% CI
Renal/Genitourinary	Dysuria	460	3	0.224		
Renal/Genitourinary	Renal failure	261	4	0.127	0.001	114.63 [31.31–419.68]
Renal/Genitourinary	Renal function test abnormal	25	1	0.012		
Renal/Genitourinary	Renal impairment	192	1	0.093		
Renal/Genitourinary	Renal infarct	41		0.020		
Renal/Genitourinary	Specific gravity urine abnormal	3				
Renal/Genitourinary	Urine abnormality	79	3	0.038		
Renal/Genitourinary	Urine analysis abnormal	307	3	0.149		
Renal/Genitourinary	Urine odour abnormal	86		0.042		
Renal/Genitourinary	Biopsy adrenal gland abnormal	1				
Renal/Genitourinary	Foetal renal impairment	2				
Renal/Genitourinary	Renal scan abnormal	1				
Renal/Genitourinary	pH urine abnormal	1				
Renal/Genitourinary	Prerenal failure	1				
Sexual organs	Abnormal uterine bleeding	70		0.034		
Sexual organs	Abortion	3				
Sexual organs	Abortion complete	1				
Sexual organs	Abortion induced	22		0.011		
Sexual organs	Abortion missed	22		0.011		
Sexual organs	Abortion of ectopic pregnancy	1				
Sexual organs	Abortion spontaneous	870	9	0.423	0.002	169.83 [71.65–402.55]
Sexual organs	Abortion spontaneous incomplete	1				
Sexual organs	Abortion threatened	16		0.008		
Sexual organs	Adnexa uteri pain	87		0.042		
Sexual organs	Amenorrhoea	400	1	0.195		
Sexual organs	Erection increased	7		0.003		
Sexual organs	Heavy menstrual bleeding	3221		1.568		
Sexual organs	Infertility	1				
Sexual organs	Infertility female	6		0.003		
Sexual organs	Infertility tests	1				
Sexual organs	Intermenstrual bleeding	982		0.478		
Sexual organs	Menstrual disorder	1844	2	0.897		
Sexual organs	Penile haemorrhage	5		0.002		
Sexual organs	Penile pain	10		0.005		
Sexual organs	Postmenopausal haemorrhage	385	1	0.187		
Sexual organs	Premature menarche	3				
Sexual organs	Premenstrual pain	36		0.018		

Continues on the next page ... . . .

CTC	Reaction	COVID	Influenza	R_c	R_n	RR 99% CI
Sexual organs	Premenstrual syndrome	112		0.055		
Sexual organs	Priapism	5		0.002		
Sexual organs	Spontaneous penile erection	5		0.002		
Sexual organs	Testicular pain	155	1	0.075		
Sexual organs	Testicular swelling	76	1	0.037		
Sexual organs	Uterine haemorrhage	61		0.030		
Sexual organs	Uterine pain	42		0.020		
Sexual organs	Vaginal discharge	100	1	0.049		
Sexual organs	Vaginal haemorrhage	1183	1	0.576		
Sexual organs	Vulvovaginal dryness	14		0.007		
Sexual organs	Vulvovaginal pain	76		0.037		
Sexual organs	Abortion spontaneous complete	1				
Sexual organs	Induced abortion failed	1				
Sexual organs	Post abortion haemorrhage	1				
Thrombosis	Aortic thrombosis	19		0.009		
Thrombosis	Arterial thrombosis	25		0.012		
Thrombosis	Arteriovenous fistula thrombosis	1				
Thrombosis	Atrial thrombosis	9		0.004		
Thrombosis	Axillary vein thrombosis	17		0.008		
Thrombosis	Basilar artery thrombosis	10		0.005		
Thrombosis	Brachiocephalic vein thrombosis	8		0.004		
Thrombosis	Brain stem thrombosis	8		0.004		
Thrombosis	Cardiac ventricular thrombosis	24		0.012		
Thrombosis	Carotid artery thrombosis	23		0.011		
Thrombosis	Catheter site thrombosis	1				
Thrombosis	Cavernous sinus thrombosis	6		0.003		
Thrombosis	Cerebellar artery thrombosis	1				
Thrombosis	Cerebral artery thrombosis	17		0.008		
Thrombosis	Cerebral thrombosis	141		0.069		
Thrombosis	Cerebral venous sinus thrombosis	157		0.076		
Thrombosis	Cerebral venous thrombosis	37		0.018		
Thrombosis	Coronary artery thrombosis	41		0.020		
Thrombosis	Deep vein thrombosis	1884		0.917		
Thrombosis	Foetal placental thrombosis	6		0.003		
Thrombosis	Hepatic artery thrombosis	5		0.002		
Thrombosis	Hepatic vein thrombosis	11		0.005		
Thrombosis	Injection site thrombosis	7		0.003		

Continues on the next page ... . . .

CTC	Reaction	COVID	Influenza	R_c	R_n	RR 99% CI
Thrombosis	Jugular vein thrombosis	45		0.022		
Thrombosis	Mesenteric artery thrombosis	6		0.003		
Thrombosis	Mesenteric vein thrombosis	45		0.022		
Thrombosis	Ophthalmic artery thrombosis	4		0.002		
Thrombosis	Ophthalmic vein thrombosis	3	1			
Thrombosis	Ovarian vein thrombosis	12		0.006		
Thrombosis	Pelvic venous thrombosis	26		0.013		
Thrombosis	Penile vein thrombosis	1				
Thrombosis	Peripheral artery thrombosis	56		0.027		
Thrombosis	Portal vein thrombosis	65		0.032		
Thrombosis	Post thrombotic syndrome	3				
Thrombosis	Postoperative thrombosis	3				
Thrombosis	Postpartum venous thrombosis	1				
Thrombosis	Pulmonary artery thrombosis	13		0.006		
Thrombosis	Pulmonary thrombosis	449		0.219		
Thrombosis	Renal artery thrombosis	9		0.004		
Thrombosis	Renal vascular thrombosis	4		0.002		
Thrombosis	Renal vein thrombosis	9		0.004		
Thrombosis	Retinal artery thrombosis	5		0.002		
Thrombosis	Retinal vascular thrombosis	8		0.004		
Thrombosis	Retinal vein thrombosis	7		0.003		
Thrombosis	Splenic artery thrombosis	4		0.002		
Thrombosis	Splenic thrombosis	9		0.004		
Thrombosis	Splenic vein thrombosis	17		0.008		
Thrombosis	Subclavian artery thrombosis	4		0.002		
Thrombosis	Subclavian vein thrombosis	30		0.015		
Thrombosis	Superficial vein thrombosis	33		0.016		
Thrombosis	Superior sagittal sinus thrombosis	36		0.018		
Thrombosis	Thrombosis	2744	4	1.335	0.001	1205.20 [332.14–4373.20]
Thrombosis	Thrombosis mesenteric vessel	2				
Thrombosis	Thrombosis prophylaxis	1				
Thrombosis	Thrombotic microangiopathy	3				
Thrombosis	Thrombotic stroke	13		0.006		
Thrombosis	Thrombotic thrombocytopenic purpura	46		0.022		
Thrombosis	Transverse sinus thrombosis	50		0.024		
Thrombosis	Vaccination site thrombosis	1				
Thrombosis	Vascular access site thrombosis	1				

Continues on the next page ... . . .

CTC	Reaction	COVID	Influenza	R_c	R_n	RR 99% CI
Thrombosis	Vena cava thrombosis	12		0.006		
Thrombosis	Venous thrombosis	31	1	0.015		
Thrombosis	Venous thrombosis limb	29		0.014		
Thrombosis	Graft thrombosis	1				
Thrombosis	Hepatic vascular thrombosis	1				
Thrombosis	Intrapericardial thrombosis	1				
Thrombosis	Portosplenomesenteric venous thrombosis	3				
Thrombosis	Prosthetic cardiac valve thrombosis	2				
Thrombosis	Pulmonary venous thrombosis	4		0.002		
Thrombosis	Thrombosis in device	1				
Thrombosis	Thrombotic cerebral infarction	3				
Thrombosis	Vascular stent thrombosis	6		0.003		
Thrombosis	Infective thrombosis	1				
Thrombosis	Spinal artery thrombosis	1				
Thrombosis	Thrombosis with thrombocytopenia syndrome	2				
Thrombosis	Vascular graft thrombosis	2				
Thrombosis	Vertebral artery thrombosis	3				
Thrombosis	Visceral venous thrombosis	2				
Thrombosis	Postpartum thrombosis	1				
Thrombosis	Truncus coeliacus thrombosis	1				
Thrombosis	Umbilical cord thrombosis	1				
